# Correction: Fish community composition in the tropical archipelago of São Tomé and Príncipe

**DOI:** 10.1371/journal.pone.0329768

**Published:** 2025-08-04

**Authors:** Guillermo Porriños, Kristian Metcalfe, Ana Nuno, Manuel da Graça, Katy Walker, Adam Dixon, Márcio Guedes, Lodney Nazaré, Albertino dos Santos, Liliana P. Colman, Jemima Dimbleby, Marta Garcia-Doce, Annette C. Broderick, Brendan J. Godley, Tiago Capela Lourenço, Luisa Madruga, Hugulay Albuquerque Maia, Berry Mulligan, Philip D. Doherty

The authors, Guillermo Porriños and Philip D. Doherty, should be noted as shared co-corresponding authors on this work. Philip D. Doherty email address is: p.doherty@exeter.ac.uk
