# Supplementary figures and images for: Fish community composition in the tropical archipelago of São Tomé and Príncipe
Source: PLoS One. 2024 Nov 1;19(11):e0312849. doi: 10.1371/journal.pone.0312849 (PMC11530061; doi:10.1371/journal.pone.0312849)

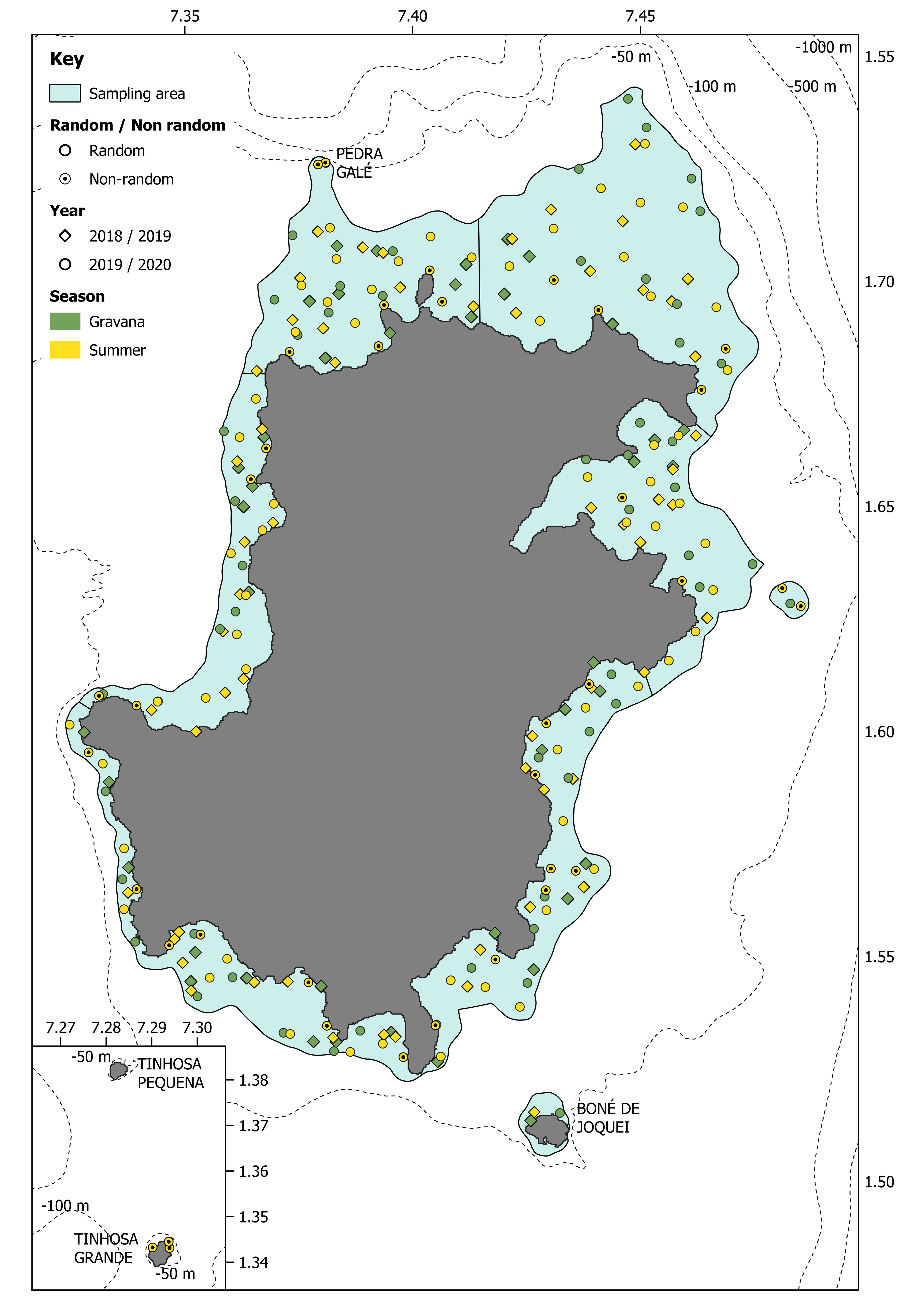

Supplement: S1 Fig — Map illustrating: (1) sampling area; (2) type of sampling (random / non-random); (3) year (2018/2019 or 209/2020); and (4) season (“gravana”, June to September; “summer”, December to February). Note that, for Príncipe’s 2019 / 2020 “summer” deployments, COVID-19 restrictions forced to delay sampling (March to July 2020), effectively entering the gravana season for that sampling round (Bathymetric data: © GEBCO). (TIF) [file pone.0312849.s001.tif]

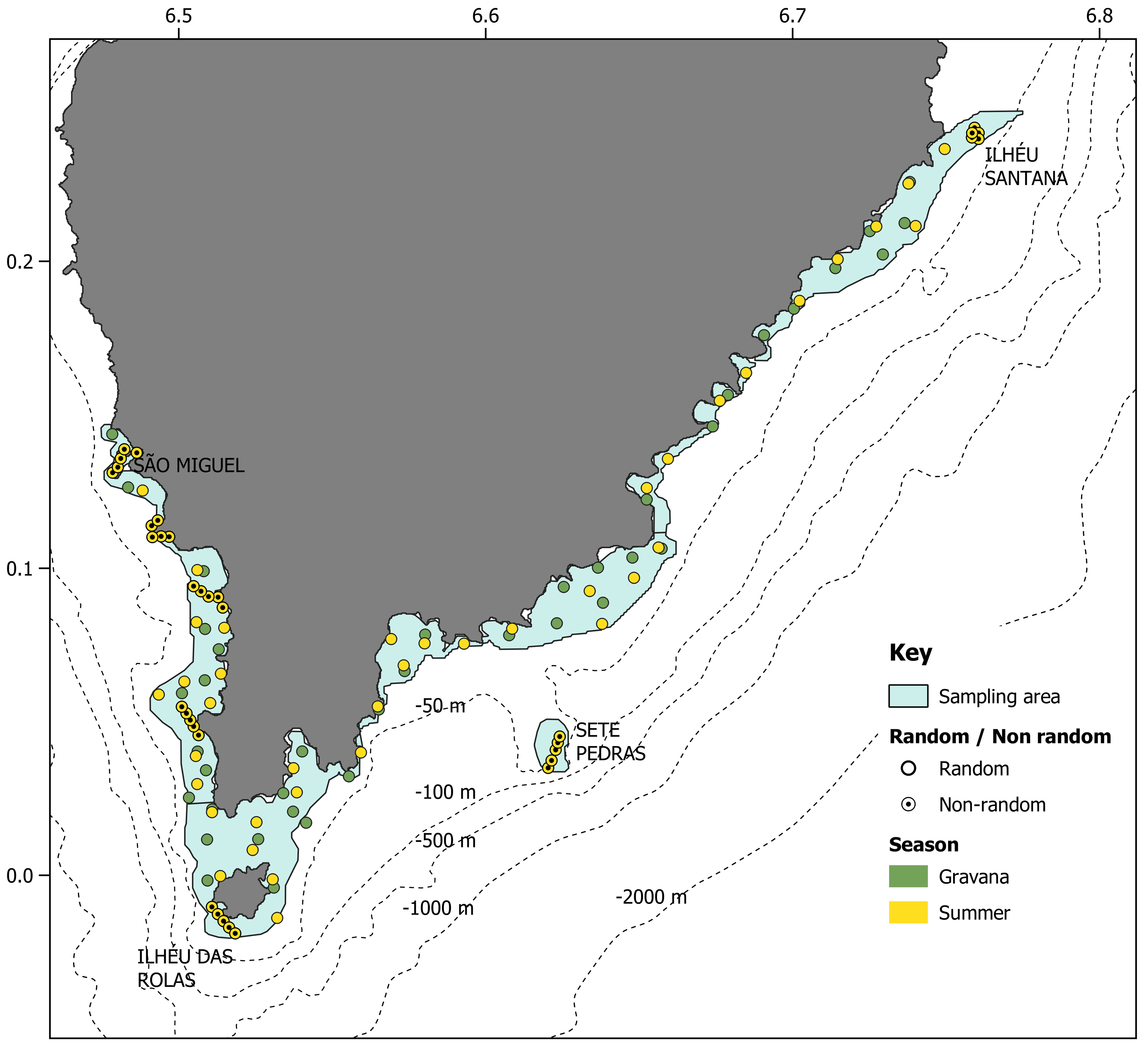

Supplement: S2 Fig — Map illustrating: (1) sampling area; (2) type of sampling (random / non-random); and (3) season (“gravana”, June to September; “summer”, December to February) (Bathymetric data: © GEBCO). (TIF) [file pone.0312849.s002.tif]

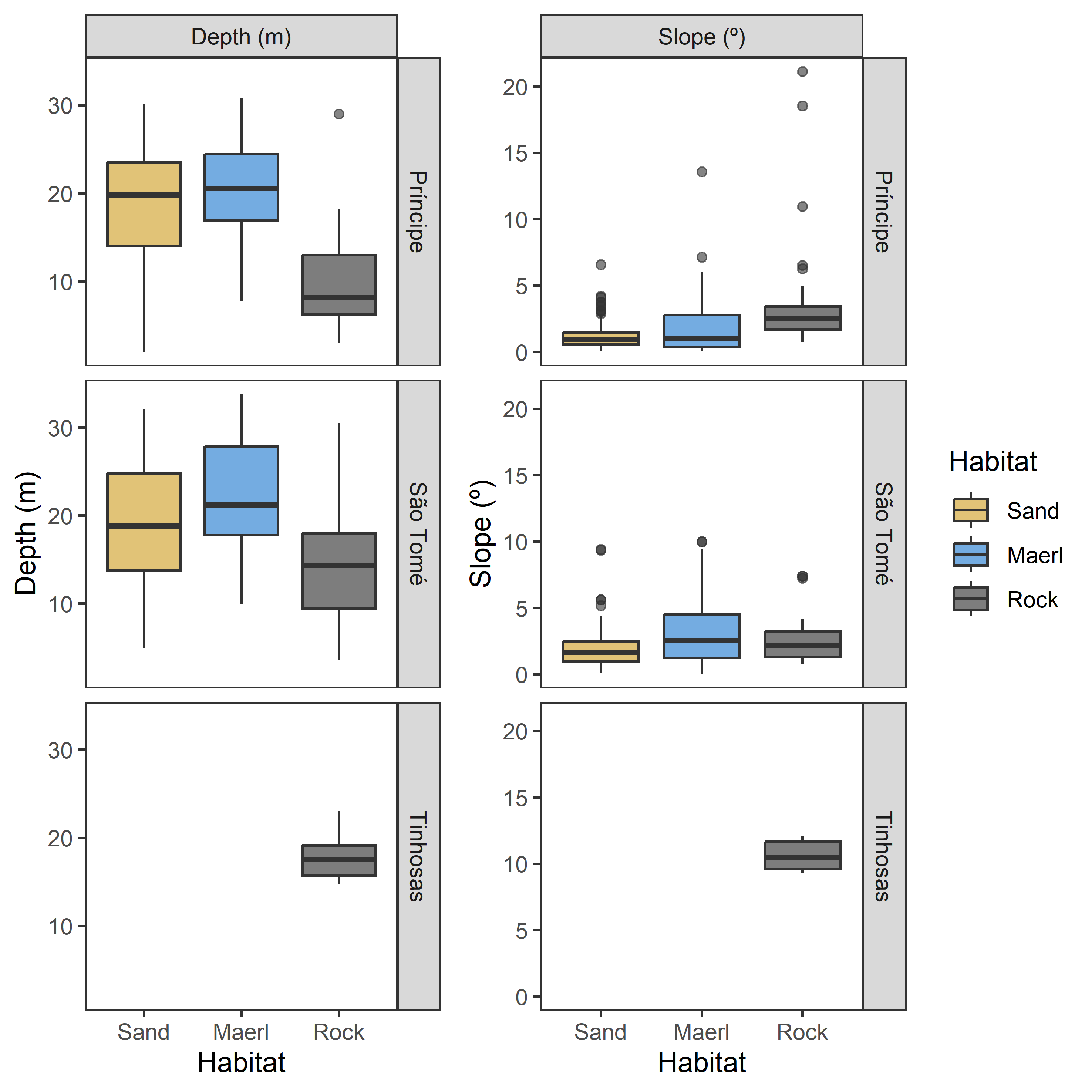

Supplement: S3 Fig — (TIF) [file pone.0312849.s003.tif]

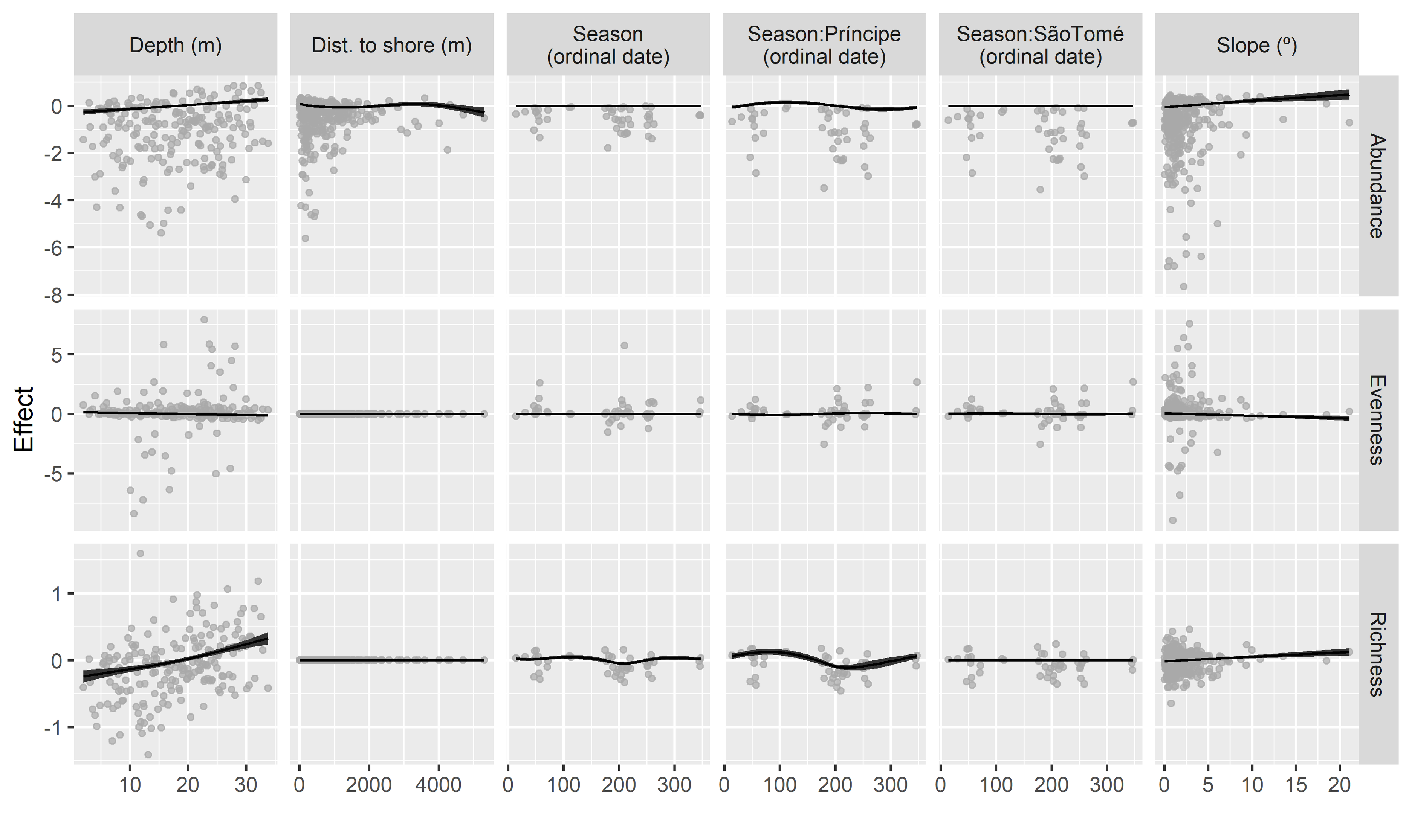

Supplement: S4 Fig — Partial effects and residuals were estimated using the R package “gratia” [47]. (TIF) [file pone.0312849.s004.tif]

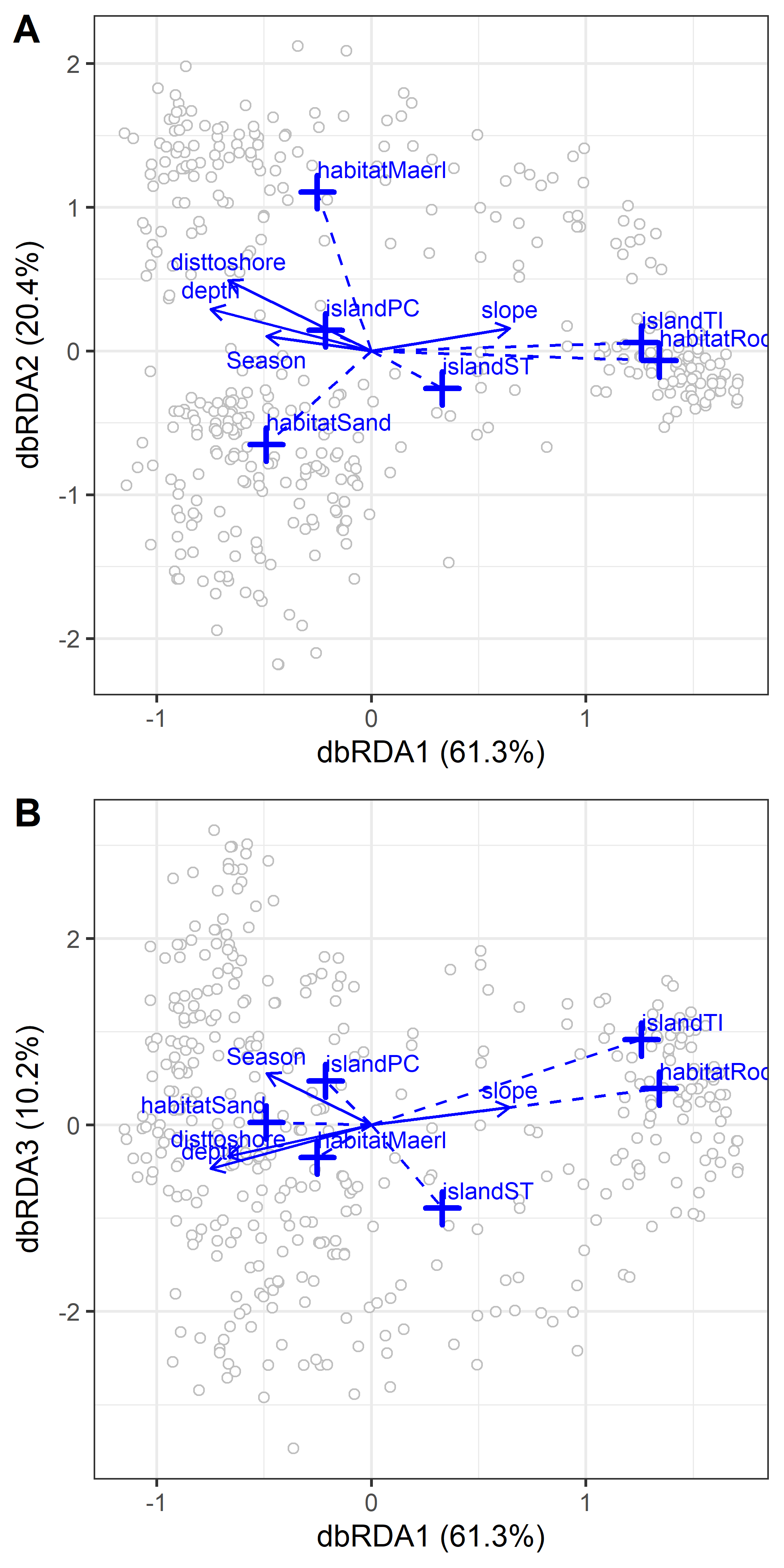

Supplement: S5 Fig — Arrows represent the coefficients of each variable on the constrained dbRDA axes, and crosses and dashed lines represent the centroid of factors in the ordination. The length of the arrow and dashed lines represent the strength of the effect of a variable or factor on community composition, and the direction (equal or opposite) indicates whether variables have positive or negative effects on the variation represented by constrained axes. See also S8 Table containing coefficients and centroids of each variable in the dbRDA ordination. (TIF) [file pone.0312849.s005.tif]

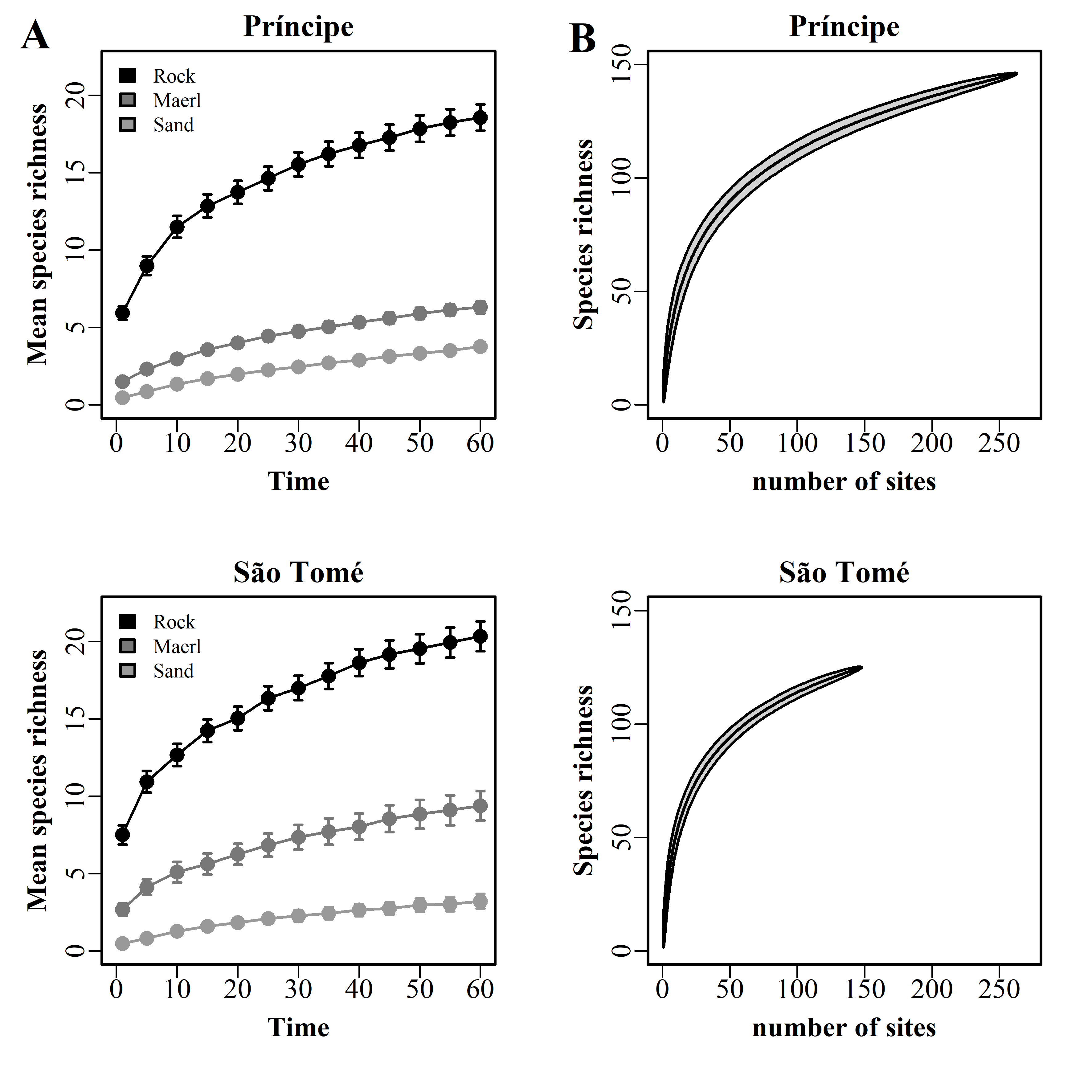

Supplement: S6 Fig — A) Mean species richness at 5-min intervals was calculated across total soak time to evaluate optimal recording times. Plots show cumulative means of species richness at different soaking times with standard error bars, disaggregated by island and habitat type. Mean species richness increased with soak time duration, with on average, 90% of species observed following 45 minutes of deployment (all habitats and islands combined). B) To assess whether sampling effort (number of sites) accurately captured species richness rarefaction curves were generated by randomly adding sites across 100 permutations using R package vegan (Oksanen et al., 2022). Plots show species accumulation plots for Príncipe and São Tomé where number of sites = BRUV deployments. (TIF) [file pone.0312849.s006.tif]
