# Supplementary material for: Fish community composition in the tropical archipelago of São Tomé and Príncipe
Source: PLoS One. 2024 Nov 1;19(11):e0312849. doi: 10.1371/journal.pone.0312849 (PMC11530061; doi:10.1371/journal.pone.0312849)
Supplement: S1 Table — (DOCX) [file pone.0312849.s007.docx]

**S1 Table**: Sampling effort per island and per sampling period.

| **Location** | **Season^1^** | **DEPLOYMENTS** | | | **Retained deployments per TYPE OF SAMPLING^4^** | | **Retained deployments per HABITAT** | | | **DATA COLLECTION** | | |
| --- | --- | --- | --- | --- | --- | --- | --- | --- | --- | --- | --- | --- |
|  |  | **Total** | **Excluded^3^** | **Retained** | **Random** | **Non random** | **Rock** | **Maerl** | **Sand** | **Start** | **End** | **Sampling days** |
| **Príncipe Island** |  | 326 | 63 | 263 | 229 | 34 | 51 | 79 | 133 | 16/07/2018 | 06/07/2020 | 37 |
|  | Gravana (2018) | 60 | 13 | 47 | 47 | 0 | 9 | 8 | 30 | 16/07/2018 | 10/08/2018 | 11 |
|  | Summer (2018/2019) | 76 | 17 | 59 | 59 | 0 | 6 | 19 | 34 | 04/12/2018 | 01/02/2019 | 7 |
|  | Gravana (2019) | 75 | 13 | 62 | 62 | 0 | 5 | 26 | 31 | 06/07/2019 | 21/09/2019 | 10 |
|  | Summer (2020)**^2^** | 66 | 12 | 54 | 37 | 17 | 15 | 22 | 17 | 12/03/2020 | 24/04/2020 | 4 |
|  | Gravana (2020)**^2^** | 49 | 8 | 41 | 24 | 17 | 16 | 4 | 21 | 24/06/2020 | 06/07/2020 | 5 |
| **Tinhosas Islets** |  | 9 | 3 | 6 | 0 | 6 | 6 | 0 | 0 | 01/12/2018 | 03/03/2020 | 3 |
|  | Summer (2018/2019) | 3 | 3 | 0 | 0 | 0 | 0 | 0 | 0 | 01/12/2018 | 01/12/2018 | 1 |
|  | Gravana (2019) | 3 | 0 | 3 | 0 | 3 | 0 | 0 | 0 | 12/07/2019 | 12/07/2019 | 1 |
|  | Summer (2019/2020) | 3 | 0 | 3 | 0 | 3 | 0 | 0 | 0 | 03/03/2020 | 03/03/2020 | 1 |
| **São Tomé Island** |  | 163 | 15 | 148 | 79 | 69 | 36 | 41 | 71 | 06/09/2019 | 27/02/2020 | 16 |
|  | Gravana (2019) | 83 | 9 | 74 | 40 | 34 | 15 | 22 | 37 | 06/09/2019 | 17/09/2019 | 8 |
|  | Summer (2019/2020) | 80 | 6 | 74 | 39 | 35 | 21 | 19 | 34 | 17/02/2020 | 27/02/2020 | 8 |

**Table notes**: (1) Sampling periods are representative of the colder months of “gravana” (June to September) and the warmer months of the summer (December to March); (2) The fourth sampling period on Príncipe (which started on the 2019/2020 summer) was delayed by disruptions caused by COVID-19, effectively entering the colder months of gravana; (3) Deployments excluded from analysis due to data loss, camera/battery failure, low visibility and loss of BRUVS; (4) BRUVS deployment locations were selected by a combination of random and non-random sampling, with some locations chosen deliberately to increase representation of rocky habitats (see section 2.2 of main article and S1.1 of Supplementary Material).
