# Supplementary material for: Fish community composition in the tropical archipelago of São Tomé and Príncipe
Source: PLoS One. 2024 Nov 1;19(11):e0312849. doi: 10.1371/journal.pone.0312849 (PMC11530061; doi:10.1371/journal.pone.0312849)
Supplement: S2 Table — (DOCX) [file pone.0312849.s008.docx]

**S2 Table**: Summary of environmental conditions of deployments.

| **Variable** | **Island** | **Max** | **Min** | **Mean** | **SD** |
| --- | --- | --- | --- | --- | --- |
| **Depth** | Principe | 30.8 | 2.0 | 17.5 | 6.9 |
|  | Tinhosas | 23.0 | 14.7 | 17.9 | 3.0 |
|  | São Tomé | 33.8 | 3.6 | 18.8 | 7.1 |
| **Dist. to shore** | Principe | 5311.5 | 6.5 | 756.5 | 852.8 |
|  | Tinhosas | 76.5 | 35.0 | 57.0 | 18.6 |
|  | São Tomé | 2050.4 | 22.8 | 403.8 | 370.1 |
| **Slope** | Principe | 21.1 | 0.0 | 1.8 | 2.3 |
|  | Tinhosas | 12.1 | 9.3 | 10.6 | 1.2 |
|  | São Tomé | 10.0 | 0.0 | 2.6 | 2.1 |
