# Supplementary material for: Fish community composition in the tropical archipelago of São Tomé and Príncipe
Source: PLoS One. 2024 Nov 1;19(11):e0312849. doi: 10.1371/journal.pone.0312849 (PMC11530061; doi:10.1371/journal.pone.0312849)
Supplement: S3 Table — (DOCX) [file pone.0312849.s009.docx]

**S3 Table**: Species’ occurrences (expressed as counts and as percentages of deployments), total MaxN across deployments, mean MaxN per deployment and mean MaxN per occurrence.

| **Family** | **Type** | **Species** | **Príncipe** | | | | | **São Tomé** | | | | | **Tinhosas** | | | | |
| --- | --- | --- | --- | --- | --- | --- | --- | --- | --- | --- | --- | --- | --- | --- | --- | --- | --- |
|  |  |  | **Occ.** | **Occ. (% depl.)** | **MaxN total** | **Mean MaxN per depl.** | **Mean MaxN per occ.** | **Occ.** | **Occ. (% depl.)** | **MaxN total** | **Mean MaxN per depl.** | **Mean MaxN per occ.** | **Occ.** | **Occ. (% depl.)** | **MaxN total** | **Mean MaxN per depl.** | **Mean MaxN per occ.** |
| Carcharhinidae | Elasmobranch | *Carcharhinus galapagensis* | 1 | 0.4% | 2 | 0.008 | 2.0 | 1 | 0.7% | 1 | 0.007 | 1.0 | 0 | 0.0% | 0 | 0.000 | NA |
| Carcharhinidae | Elasmobranch | *Carcharhinus limbatus* | 2 | 0.8% | 2 | 0.008 | 1.0 | 0 | 0.0% | 0 | 0.000 | NA | 0 | 0.0% | 0 | 0.000 | NA |
| Carcharhinidae | Elasmobranch | *Negaprion brevirostris* | 1 | 0.4% | 4 | 0.015 | 4.0 | 0 | 0.0% | 0 | 0.000 | NA | 0 | 0.0% | 0 | 0.000 | NA |
| Dasyatidae | Elasmobranch | *Hypanus rudis* | 13 | 5.0% | 15 | 0.057 | 1.15 | 4 | 2.8% | 4 | 0.028 | 1.0 | 0 | 0.0% | 0 | 0.000 | NA |
| Dasyatidae | Elasmobranch | *Taeniurops grabatus* | 5 | 1.9% | 6 | 0.023 | 1.2 | 0 | 0.0% | 0 | 0.000 | NA | 0 | 0.0% | 0 | 0.000 | NA |
| Ginglymostomatidae | Elasmobranch | *Ginglymostoma cirratum* | 3 | 1.2% | 4 | 0.015 | 1.3 | 0 | 0.0% | 0 | 0.000 | NA | 0 | 0.0% | 0 | 0.000 | NA |
| Mobulidae | Elasmobranch | *Mobula sp.* | 1 | 0.4% | 1 | 0.004 | 1.0 | 1 | 0.7% | 1 | 0.007 | 1.0 | 0 | 0.0% | 0 | 0.000 | NA |
| Sphyrnidae | Elasmobranch | *Sphyrna lewini* | 0 | 0.0% | 0 | 0.000 | NA | 5 | 3.5% | 5 | 0.035 | 1.0 | 0 | 0.0% | 0 | 0.000 | NA |
| Acanthuridae | Teleost | *Acanthurus monroviae* | 58 | 22.3% | 257 | 0.988 | 4.4 | 45 | 31.3% | 163 | 1.132 | 3.6 | 4 | 66.7% | 17 | 2.833 | 4.3 |
| Acanthuridae | Teleost | *Prionurus biafraensis* | 19 | 7.3% | 651 | 2.504 | 34.3 | 13 | 9.0% | 59 | 0.410 | 4.5 | 1 | 16.7% | 1 | 0.167 | 1.0 |
| Apogonidae | Teleost | *Phaeoptyx pigmentaria* | 1 | 0.4% | 1 | 0.004 | 1.0 | 0 | 0.0% | 0 | 0.000 | NA | 0 | 0.0% | 0 | 0.000 | NA |
| Atherinidae | Teleost | *Atherina lopeziana* | 1 | 0.4% | 2 | 0.008 | 2.0 | 0 | 0.0% | 0 | 0.000 | NA | 0 | 0.0% | 0 | 0.000 | NA |
| Aulostomidae | Teleost | *Aulostomus strigosus* | 4 | 1.5% | 4 | 0.015 | 1.0 | 23 | 16.0% | 38 | 0.264 | 1.7 | 3 | 50.0% | 8 | 1.333 | 2.7 |
| Balistidae | Teleost | *Balistes capriscus* | 19 | 7.3% | 37 | 0.142 | 1.9 | 10 | 6.9% | 11 | 0.076 | 1.1 | 0 | 0.0% | 0 | 0.000 | NA |
| Balistidae | Teleost | *Balistes punctatus* | 67 | 25.8% | 114 | 0.438 | 1.7 | 46 | 31.9% | 65 | 0.451 | 1.4 | 3 | 50.0% | 3 | 0.500 | 1.0 |
| Balistidae | Teleost | *Canthidermis sufflamen* | 5 | 1.9% | 7 | 0.027 | 1.4 | 1 | 0.7% | 2 | 0.014 | 2.0 | 4 | 66.7% | 6 | 1.000 | 1.5 |
| Balistidae | Teleost | *Melichthys niger* | 0 | 0.0% | 0 | 0.000 | NA | 4 | 2.8% | 9 | 0.063 | 2.3 | 3 | 50.0% | 28 | 4.667 | 9.3 |
| Belonidae | Teleost | *Ablennes hians* | 0 | 0.0% | 0 | 0.000 | NA | 1 | 0.7% | 1 | 0.007 | 1.0 | 0 | 0.0% | 0 | 0.000 | NA |
| Blenniidae | Teleost | *Hypleurochilus aequipinnis* | 1 | 0.4% | 3 | 0.012 | 3.0 | 0 | 0.0% | 0 | 0.000 | NA | 0 | 0.0% | 0 | 0.000 | NA |
| Blenniidae | Teleost | *Microlipophrys velifer* | 0 | 0.0% | 0 | 0.000 | NA | 1 | 0.7% | 1 | 0.007 | 1.0 | 0 | 0.0% | 0 | 0.000 | NA |
| Blenniidae | Teleost | *Ophioblennius atlanticus* | 8 | 3.1% | 8 | 0.031 | 1.0 | 8 | 5.6% | 17 | 0.118 | 2.1 | 2 | 33.3% | 3 | 0.500 | 1.5 |
| Bothidae | Teleost | *Bothus guibei* | 1 | 0.4% | 1 | 0.004 | 1.0 | 0 | 0.0% | 0 | 0.000 | NA | 0 | 0.0% | 0 | 0.000 | NA |
| Bothidae | Teleost | *Bothus lunatus* | 0 | 0.0% | 0 | 0.000 | NA | 2 | 1.4% | 4 | 0.028 | 2.0 | 0 | 0.0% | 0 | 0.000 | NA |
| Bothidae | Teleost | *Bothus podas* | 1 | 0.4% | 1 | 0.004 | 1.0 | 0 | 0.0% | 0 | 0.000 | NA | 0 | 0.0% | 0 | 0.000 | NA |
| Carangidae | Teleost | *Alectis alexandrina* | 1 | 0.4% | 1 | 0.004 | 1.0 | 4 | 2.8% | 4 | 0.028 | 1.0 | 0 | 0.0% | 0 | 0.000 | NA |
| Carangidae | Teleost | *Alectis ciliaris* | 4 | 1.5% | 5 | 0.019 | 1.3 | 3 | 2.1% | 4 | 0.028 | 1.3 | 0 | 0.0% | 0 | 0.000 | NA |
| Carangidae | Teleost | *Caranx bartholomaei* | 127 | 48.8% | 433 | 1.665 | 3.4 | 33 | 22.9% | 133 | 0.924 | 4.0 | 6 | 100.0% | 17 | 2.833 | 2.8 |
| Carangidae | Teleost | *Caranx crysos* | 157 | 60.4% | 453 | 1.742 | 2.9 | 32 | 22.2% | 84 | 0.583 | 2.6 | 5 | 83.3% | 10 | 1.667 | 2.0 |
| Carangidae | Teleost | *Caranx fischeri* | 2 | 0.8% | 2 | 0.008 | 1.0 | 0 | 0.0% | 0 | 0.000 | NA | 0 | 0.0% | 0 | 0.000 | NA |
| Carangidae | Teleost | *Caranx hippos* | 26 | 10.0% | 35 | 0.135 | 1.3 | 9 | 6.3% | 46 | 0.319 | 5.1 | 0 | 0.0% | 0 | 0.000 | NA |
| Carangidae | Teleost | *Caranx latus* | 5 | 1.9% | 14 | 0.054 | 2.8 | 1 | 0.7% | 1 | 0.007 | 1.0 | 0 | 0.0% | 0 | 0.000 | NA |
| Carangidae | Teleost | *Caranx lugubris* | 8 | 3.1% | 9 | 0.035 | 1.1 | 0 | 0.0% | 0 | 0.000 | NA | 0 | 0.0% | 0 | 0.000 | NA |
| Carangidae | Teleost | *Decapterus macarellus* | 14 | 5.4% | 784 | 3.015 | 56.0 | 3 | 2.1% | 125 | 0.868 | 41.7 | 0 | 0.0% | 0 | 0.000 | NA |
| Carangidae | Teleost | *Decapterus punctatus* | 2 | 0.8% | 7 | 0.027 | 3.5 | 6 | 4.2% | 182 | 1.264 | 30.3 | 0 | 0.0% | 0 | 0.000 | NA |
| Carangidae | Teleost | *Elagatis bipinnulata* | 18 | 6.9% | 24 | 0.092 | 1.3 | 2 | 1.4% | 2 | 0.014 | 1.0 | 0 | 0.0% | 0 | 0.000 | NA |
| Carangidae | Teleost | *Selar crumenopthalmus* | 0 | 0.0% | 0 | 0.000 | NA | 0 | 0.0% | 0 | 0.000 | NA | 0 | 0.0% | 0 | 0.000 | NA |
| Carangidae | Teleost | *Seriola rivoliana* | 10 | 3.8% | 11 | 0.042 | 1.1 | 0 | 0.0% | 0 | 0.000 | NA | 1 | 16.7% | 1 | 0.167 | 1.0 |
| Carangidae | Teleost | *Trachinotus ovatus* | 3 | 1.2% | 35 | 0.135 | 11.7 | 3 | 2.1% | 9 | 0.063 | 3.0 | 0 | 0.0% | 0 | 0.000 | NA |
| Chaetodontidae | Teleost | *Chaetodon robustus* | 4 | 1.5% | 6 | 0.023 | 1.5 | 21 | 14.6% | 37 | 0.257 | 1.8 | 0 | 0.0% | 0 | 0.000 | NA |
| Cirrhitidae | Teleost | *Cirrhitus atlanticus* | 26 | 10.0% | 32 | 0.123 | 1.2 | 17 | 11.8% | 25 | 0.174 | 1.5 | 4 | 66.7% | 5 | 0.833 | 1.3 |
| Clupeidae | Teleost | *Sardinella sp.* | 2 | 0.8% | 24 | 0.092 | 12.0 | 0 | 0.0% | 0 | 0.000 | NA | 0 | 0.0% | 0 | 0.000 | NA |

**S3 Table (cont.)**

| **Family** | **Type** | **Species** | **Príncipe** | | | | | **São Tomé** | | | | | **Tinhosas** | | | | |
| --- | --- | --- | --- | --- | --- | --- | --- | --- | --- | --- | --- | --- | --- | --- | --- | --- | --- |
|  |  |  | **Occ.** | **Occ. (% depl.)** | **MaxN total** | **Mean MaxN per depl.** | **Mean MaxN per occ.** | **Occ.** | **Occ. (% depl.)** | **MaxN total** | **Mean MaxN per depl.** | **Mean MaxN per occ.** | **Occ.** | **Occ. (% depl.)** | **MaxN total** | **Mean MaxN per depl.** | **Mean MaxN per occ.** |
| Congridae | Teleost | *Ariosoma balearicum* | 3 | 1.2% | 14 | 0.054 | 4.7 | 0 | 0.0% | 0 | 0.000 | NA | 0 | 0.0% | 0 | 0.000 | NA |
| Congridae | Teleost | *Heteroconger longissimus* | 9 | 3.5% | 117 | 0.450 | 13.0 | 1 | 0.7% | 47 | 0.326 | 47.0 | 0 | 0.0% | 0 | 0.000 | NA |
| Congridae | Teleost | *Paraconger macrops* | 2 | 0.8% | 2 | 0.008 | 1.0 | 0 | 0.0% | 0 | 0.000 | NA | 0 | 0.0% | 0 | 0.000 | NA |
| Dactylopteridae | Teleost | *Dactylopterus volitans* | 60 | 23.1% | 84 | 0.323 | 1.4 | 33 | 22.9% | 60 | 0.417 | 1.8 | 0 | 0.0% | 0 | 0.000 | NA |
| Diodontidae | Teleost | *Chilomycterus reticulatus* | 1 | 0.4% | 1 | 0.004 | 1.0 | 2 | 1.4% | 2 | 0.014 | 1.0 | 0 | 0.0% | 0 | 0.000 | NA |
| Diodontidae | Teleost | *Chilomycterus spinosus mauretanicus* | 6 | 2.3% | 6 | 0.023 | 1.0 | 8 | 5.6% | 8 | 0.056 | 1.0 | 0 | 0.0% | 0 | 0.000 | NA |
| Diodontidae | Teleost | *Diodon holocanthus* | 10 | 3.8% | 10 | 0.038 | 1.0 | 0 | 0.0% | 0 | 0.000 | NA | 0 | 0.0% | 0 | 0.000 | NA |
| Diodontidae | Teleost | *Diodon hystrix* | 5 | 1.9% | 5 | 0.019 | 1.0 | 4 | 2.8% | 4 | 0.028 | 1.0 | 0 | 0.0% | 0 | 0.000 | NA |
| Echeneidae | Teleost | *Echeneis naucrates* | 9 | 3.5% | 18 | 0.069 | 2.0 | 7 | 4.9% | 8 | 0.056 | 1.1 | 1 | 16.7% | 1 | 0.167 | 1.0 |
| Ephippidae | Teleost | *Ephippus goreensis* | 0 | 0.0% | 0 | 0.000 | NA | 1 | 0.7% | 1 | 0.007 | 1.0 | 0 | 0.0% | 0 | 0.000 | NA |
| Fistulariidae | Teleost | *Fistularia tabacaria* | 3 | 1.2% | 3 | 0.012 | 1.0 | 21 | 14.6% | 29 | 0.201 | 1.4 | 0 | 0.0% | 0 | 0.000 | NA |
| Gerreidae | Teleost | *Eucinostomus melanopterus* | 0 | 0.0% | 0 | 0.000 | NA | 1 | 0.7% | 2 | 0.014 | 2.0 | 0 | 0.0% | 0 | 0.000 | NA |
| Gobiidae | Teleost | *Gnatholepis thompsoni* | 1 | 0.4% | 2 | 0.008 | 2.0 | 0 | 0.0% | 0 | 0.000 | NA | 0 | 0.0% | 0 | 0.000 | NA |
| Gobiidae | Teleost | *Gobius rubropunctatus* | 1 | 0.4% | 1 | 0.004 | 1.0 | 3 | 2.1% | 4 | 0.028 | 1.3 | 0 | 0.0% | 0 | 0.000 | NA |
| Haemulidae | Teleost | *Parakuhlia macrophthalmus* | 0 | 0.0% | 0 | 0.000 | NA | 0 | 0.0% | 0 | 0.000 | NA | 1 | 16.7% | 1 | 0.167 | 1.0 |
| Haemulidae | Teleost | *Pomadasys incisus* | 0 | 0.0% | 0 | 0.000 | NA | 2 | 1.4% | 21 | 0.146 | 10.5 | 0 | 0.0% | 0 | 0.000 | NA |
| Holocentridae | Teleost | *Holocentrus adscensionis* | 21 | 8.1% | 66 | 0.254 | 3.1 | 18 | 12.5% | 61 | 0.424 | 3.4 | 2 | 33.3% | 4 | 0.667 | 2.0 |
| Holocentridae | Teleost | *Myripristis jacobus* | 11 | 4.2% | 55 | 0.212 | 5.0 | 4 | 2.8% | 20 | 0.139 | 5.0 | 2 | 33.3% | 15 | 2.500 | 7.5 |
| Holocentridae | Teleost | *Sargocentron hastatum* | 0 | 0.0% | 0 | 0.000 | NA | 1 | 0.7% | 1 | 0.007 | 1.0 | 0 | 0.0% | 0 | 0.000 | NA |
| Kyphosidae | Teleost | *Kyphosus incisor* | 6 | 2.3% | 40 | 0.154 | 6.7 | 1 | 0.7% | 1 | 0.007 | 1.0 | 0 | 0.0% | 0 | 0.000 | NA |
| Kyphosidae | Teleost | *Kyphosus sectatrix* | 6 | 2.3% | 53 | 0.204 | 8.8 | 0 | 0.0% | 0 | 0.000 | NA | 0 | 0.0% | 0 | 0.000 | NA |
| Labridae | Teleost | *Bodianus pulchellus* | 3 | 1.2% | 3 | 0.012 | 1.0 | 3 | 2.1% | 4 | 0.028 | 1.3 | 4 | 66.7% | 7 | 1.167 | 1.8 |
| Labridae | Teleost | *Bodianus speciosus* | 41 | 15.8% | 67 | 0.258 | 1.6 | 34 | 23.6% | 57 | 0.396 | 1.7 | 0 | 0.0% | 0 | 0.000 | NA |
| Labridae | Teleost | *Clepticus africanus* | 6 | 2.3% | 61 | 0.235 | 10.2 | 6 | 4.2% | 113 | 0.785 | 18.8 | 0 | 0.0% | 0 | 0.000 | NA |
| Labridae | Teleost | *Coris atlantica* | 44 | 16.9% | 107 | 0.412 | 2.4 | 27 | 18.8% | 86 | 0.597 | 3.2 | 6 | 100.0% | 15 | 2.500 | 2.5 |
| Labridae | Teleost | *Thalassoma ascensionis* | 0 | 0.0% | 0 | 0.000 | NA | 0 | 0.0% | 0 | 0.000 | NA | 1 | 16.7% | 1 | 0.167 | 1.0 |
| Labridae | Teleost | *Thalassoma newtoni* | 49 | 18.8% | 160 | 0.615 | 3.3 | 55 | 38.2% | 283 | 1.965 | 5.1 | 6 | 100.0% | 46 | 7.667 | 7.7 |
| Labridae | Teleost | *Thalassoma pavo* | 1 | 0.4% | 1 | 0.004 | 1.0 | 0 | 0.0% | 0 | 0.000 | NA | 2 | 33.3% | 6 | 1.000 | 3.0 |
| Labridae | Teleost | *Xyrichtys novacula* | 76 | 29.2% | 219 | 0.842 | 2.9 | 18 | 12.5% | 60 | 0.417 | 3.3 | 0 | 0.0% | 0 | 0.000 | NA |
| Labrisomidae | Teleost | *Labrisomus nuchipinnis* | 1 | 0.4% | 1 | 0.004 | 1.0 | 4 | 2.8% | 7 | 0.049 | 1.8 | 0 | 0.0% | 0 | 0.000 | NA |
| Lethrinidae | Teleost | *Lethrinus atlanticus* | 60 | 23.1% | 287 | 1.104 | 4.8 | 29 | 20.1% | 65 | 0.451 | 2.2 | 0 | 0.0% | 0 | 0.000 | NA |
| Lutjanidae | Teleost | *Apsilus fuscus* | 1 | 0.4% | 2 | 0.008 | 2.0 | 4 | 2.8% | 10 | 0.069 | 2.5 | 0 | 0.0% | 0 | 0.000 | NA |
| Lutjanidae | Teleost | *Lutjanus agennes* | 27 | 10.4% | 74 | 0.285 | 2.7 | 9 | 6.3% | 22 | 0.153 | 2.4 | 5 | 83.3% | 57 | 9.500 | 11.4 |
| Lutjanidae | Teleost | *Lutjanus dentatus* | 12 | 4.6% | 12 | 0.046 | 1.0 | 5 | 3.5% | 5 | 0.035 | 1.0 | 2 | 33.3% | 3 | 0.500 | 1.5 |
| Lutjanidae | Teleost | *Lutjanus endecacanthus* | 4 | 1.5% | 5 | 0.019 | 1.3 | 1 | 0.7% | 1 | 0.007 | 1.0 | 0 | 0.0% | 0 | 0.000 | NA |
| Lutjanidae | Teleost | *Lutjanus fulgens* | 15 | 5.8% | 88 | 0.338 | 5.9 | 7 | 4.9% | 41 | 0.285 | 5.9 | 0 | 0.0% | 0 | 0.000 | NA |
| Lutjanidae | Teleost | *Lutjanus goreensis* | 3 | 1.2% | 3 | 0.012 | 1.0 | 6 | 4.2% | 16 | 0.111 | 2.7 | 1 | 16.7% | 1 | 0.167 | 1.0 |
| Lutjanidae | Teleost | *Lutjanus griseus* | 1 | 0.4% | 3 | 0.012 | 3.0 | 0 | 0.0% | 0 | 0.000 | NA | 0 | 0.0% | 0 | 0.000 | NA |
| Megalopidae | Teleost | *Megalops atlanticus* | 0 | 0.0% | 0 | 0.000 | NA | 1 | 0.7% | 1 | 0.007 | 1.0 | 0 | 0.0% | 0 | 0.000 | NA |
| Monacanthidae | Teleost | *Aluterus heudelotii* | 1 | 0.4% | 1 | 0.004 | 1.0 | 7 | 4.9% | 11 | 0.076 | 1.6 | 0 | 0.0% | 0 | 0.000 | NA |
| Monacanthidae | Teleost | *Aluterus scriptus* | 17 | 6.5% | 24 | 0.092 | 1.4 | 8 | 5.6% | 10 | 0.069 | 1.3 | 1 | 16.7% | 1 | 0.167 | 1.0 |
| Monacanthidae | Teleost | *Cantherhines macrocerus* | 1 | 0.4% | 1 | 0.004 | 1.0 | 0 | 0.0% | 0 | 0.000 | NA | 0 | 0.0% | 0 | 0.000 | NA |

**S3 Table (cont.)**

| **Family** | **Type** | **Species** | **Príncipe** | | | | | **São Tomé** | | | | | **Tinhosas** | | | | |
| --- | --- | --- | --- | --- | --- | --- | --- | --- | --- | --- | --- | --- | --- | --- | --- | --- | --- |
|  |  |  | **Occ.** | **Occ. (% depl.)** | **MaxN total** | **Mean MaxN per depl.** | **Mean MaxN per occ.** | **Occ.** | **Occ. (% depl.)** | **MaxN total** | **Mean MaxN per depl.** | **Mean MaxN per occ.** | **Occ.** | **Occ. (% depl.)** | **MaxN total** | **Mean MaxN per depl.** | **Mean MaxN per occ.** |
| Monacanthidae | Teleost | *Cantherhines pardalis* | 0 | 0.0% | 0 | 0.000 | NA | 3 | 2.1% | 7 | 0.049 | 2.3 | 0 | 0.0% | 0 | 0.000 | NA |
| Monacanthidae | Teleost | *Cantherhines pullus* | 57 | 21.9% | 89 | 0.342 | 1.6 | 74 | 51.4% | 452 | 3.139 | 6.1 | 5 | 83.3% | 11 | 1.833 | 2.2 |
| Monacanthidae | Teleost | *Stephanolepis hispidus* | 10 | 3.8% | 12 | 0.046 | 1.2 | 5 | 3.5% | 7 | 0.049 | 1.4 | 0 | 0.0% | 0 | 0.000 | NA |
| Mullidae | Teleost | *Mulloidichthys martinicus* | 31 | 11.9% | 193 | 0.742 | 6.2 | 24 | 16.7% | 118 | 0.819 | 4.9 | 6 | 100.0% | 39 | 6.500 | 6.5 |
| Mullidae | Teleost | *Mullus surmuletus* | 5 | 1.9% | 10 | 0.038 | 2.0 | 0 | 0.0% | 0 | 0.000 | NA | 0 | 0.0% | 0 | 0.000 | NA |
| Mullidae | Teleost | *Pseudupeneus prayensis* | 13 | 5.0% | 32 | 0.123 | 2.5 | 49 | 34.0% | 187 | 1.299 | 3.8 | 0 | 0.0% | 0 | 0.000 | NA |
| Muraenidae | Teleost | *Echidna peli* | 3 | 1.2% | 3 | 0.012 | 1.0 | 3 | 2.1% | 4 | 0.028 | 1.3 | 0 | 0.0% | 0 | 0.000 | NA |
| Muraenidae | Teleost | *Enchelycore nigricans* | 16 | 6.2% | 23 | 0.088 | 1.4 | 1 | 0.7% | 1 | 0.007 | 1.0 | 1 | 16.7% | 2 | 0.333 | 2.0 |
| Muraenidae | Teleost | *Gymnothorax afer* | 1 | 0.4% | 1 | 0.004 | 1.0 | 4 | 2.8% | 4 | 0.028 | 1.0 | 0 | 0.0% | 0 | 0.000 | NA |
| Muraenidae | Teleost | *Gymnothorax vicinus* | 4 | 1.5% | 4 | 0.015 | 1.0 | 17 | 11.8% | 26 | 0.181 | 1.5 | 0 | 0.0% | 0 | 0.000 | NA |
| Muraenidae | Teleost | *Muraena melanotis* | 4 | 1.5% | 9 | 0.035 | 2.3 | 8 | 5.6% | 10 | 0.069 | 1.3 | 0 | 0.0% | 0 | 0.000 | NA |
| Muraenidae | Teleost | *Muraena robusta* | 1 | 0.4% | 1 | 0.004 | 1.0 | 2 | 1.4% | 2 | 0.014 | 1.0 | 0 | 0.0% | 0 | 0.000 | NA |
| Ophichthidae | Teleost | *Myrichthys pardalis* | 3 | 1.2% | 3 | 0.012 | 1.0 | 1 | 0.7% | 1 | 0.007 | 1.0 | 0 | 0.0% | 0 | 0.000 | NA |
| Ophichthidae | Teleost | *Ophichthus ophis* | 4 | 1.5% | 4 | 0.015 | 1.0 | 2 | 1.4% | 3 | 0.021 | 1.5 | 0 | 0.0% | 0 | 0.000 | NA |
| Ophichthidae | Teleost | *Ophichthus rufus* | 0 | 0.0% | 0 | 0.000 | NA | 1 | 0.7% | 1 | 0.007 | 1.0 | 0 | 0.0% | 0 | 0.000 | NA |
| Ophichthidae | Teleost | *Pisodonophis semicinctus* | 0 | 0.0% | 0 | 0.000 | NA | 1 | 0.7% | 1 | 0.007 | 1.0 | 0 | 0.0% | 0 | 0.000 | NA |
| Ostraciidae | Teleost | *Acanthostracion guineensis* | 3 | 1.2% | 4 | 0.015 | 1.3 | 0 | 0.0% | 0 | 0.000 | NA | 0 | 0.0% | 0 | 0.000 | NA |
| Ostraciidae | Teleost | *Acanthostracion notacanthus* | 22 | 8.5% | 31 | 0.119 | 1.4 | 15 | 10.4% | 17 | 0.118 | 1.1 | 0 | 0.0% | 0 | 0.000 | NA |
| Pomacanthidae | Teleost | *Holacanthus africanus* | 39 | 15.0% | 67 | 0.258 | 1.7 | 20 | 13.9% | 32 | 0.222 | 1.6 | 5 | 83.3% | 10 | 1.667 | 2.0 |
| Pomacentridae | Teleost | *Abudefduf hoefleri* | 7 | 2.7% | 16 | 0.062 | 2.3 | 0 | 0.0% | 0 | 0.000 | NA | 1 | 16.7% | 1 | 0.167 | 1.0 |
| Pomacentridae | Teleost | *Abudefduf saxatilis* | 17 | 6.5% | 75 | 0.288 | 4.4 | 7 | 4.9% | 64 | 0.444 | 9.1 | 0 | 0.0% | 0 | 0.000 | NA |
| Pomacentridae | Teleost | *Abudefduf taurus* | 0 | 0.0% | 0 | 0.000 | NA | 0 | 0.0% | 0 | 0.000 | NA | 2 | 33.3% | 2 | 0.333 | 1.0 |
| Pomacentridae | Teleost | *Chromis cadenati* | 1 | 0.4% | 1 | 0.004 | 1.0 | 1 | 0.7% | 45 | 0.313 | 45.0 | 0 | 0.0% | 0 | 0.000 | NA |
| Pomacentridae | Teleost | *Chromis multilineata* | 31 | 11.9% | 832 | 3.200 | 26.8 | 37 | 25.7% | 1246 | 8.653 | 33.7 | 4 | 66.7% | 404 | 67.333 | 101.0 |
| Pomacentridae | Teleost | *Microspathodon frontatus* | 10 | 3.8% | 63 | 0.242 | 6.3 | 1 | 0.7% | 5 | 0.035 | 5.0 | 0 | 0.0% | 0 | 0.000 | NA |
| Pomacentridae | Teleost | *Stegastes imbricatus* | 20 | 7.7% | 76 | 0.292 | 3.8 | 30 | 20.8% | 85 | 0.590 | 2.8 | 3 | 50.0% | 14 | 2.333 | 4.7 |
| Priacanthidae | Teleost | *Heteropriacanthus cruentatus* | 1 | 0.4% | 1 | 0.004 | 1.0 | 2 | 1.4% | 2 | 0.014 | 1.0 | 1 | 16.7% | 3 | 0.500 | 3.0 |
| Scaridae | Teleost | *Nicholsina usta* | 0 | 0.0% | 0 | 0.000 | NA | 0 | 0.0% | 0 | 0.000 | NA | 1 | 16.7% | 2 | 0.333 | 2.0 |
| Scaridae | Teleost | *Scarus hoefleri* | 25 | 9.6% | 57 | 0.219 | 2.3 | 18 | 12.5% | 47 | 0.326 | 2.6 | 4 | 66.7% | 4 | 0.667 | 1.0 |
| Scaridae | Teleost | *Sparisoma choati* | 47 | 18.1% | 178 | 0.685 | 3.8 | 44 | 30.6% | 162 | 1.125 | 3.7 | 6 | 100.0% | 18 | 3.000 | 3.0 |
| Scaridae | Teleost | *Sparisoma rubripinne* | 4 | 1.5% | 7 | 0.027 | 1.8 | 2 | 1.4% | 3 | 0.021 | 1.5 | 2 | 33.3% | 3 | 0.500 | 1.5 |
| Scombridae | Teleost | *Acanthocybium solandri* | 1 | 0.4% | 1 | 0.004 | 1.0 | 0 | 0.0% | 0 | 0.000 | NA | 0 | 0.0% | 0 | 0.000 | NA |
| Scombridae | Teleost | *Auxis rochei* | 1 | 0.4% | 1 | 0.004 | 1.0 | 0 | 0.0% | 0 | 0.000 | NA | 0 | 0.0% | 0 | 0.000 | NA |
| Scombridae | Teleost | *Auxis thazard* | 1 | 0.4% | 1 | 0.004 | 1.0 | 2 | 1.4% | 7 | 0.049 | 3.5 | 0 | 0.0% | 0 | 0.000 | NA |
| Scombridae | Teleost | *Katsuwonus pelamis* | 1 | 0.4% | 1 | 0.004 | 1.0 | 0 | 0.0% | 0 | 0.000 | NA | 0 | 0.0% | 0 | 0.000 | NA |
| Scombridae | Teleost | *Scomber colias* | 1 | 0.4% | 1 | 0.004 | 1.0 | 0 | 0.0% | 0 | 0.000 | NA | 0 | 0.0% | 0 | 0.000 | NA |
| Scombridae | Teleost | *Scomberomorus tritor* | 11 | 4.2% | 12 | 0.046 | 1.1 | 13 | 9.0% | 15 | 0.104 | 1.2 | 0 | 0.0% | 0 | 0.000 | NA |
| Scorpaenidea | Teleost | *Scorpaena laevis* | 1 | 0.4% | 1 | 0.004 | 1.0 | 1 | 0.7% | 1 | 0.007 | 1.0 | 0 | 0.0% | 0 | 0.000 | NA |
| Serranidae | Teleost | *Cephalopholis nigri* | 38 | 14.6% | 82 | 0.315 | 2.2 | 26 | 18.1% | 42 | 0.292 | 1.6 | 0 | 0.0% | 0 | 0.000 | NA |
| Serranidae | Teleost | *Cephalopholis taeniops* | 24 | 9.2% | 55 | 0.212 | 2.3 | 39 | 27.1% | 100 | 0.694 | 2.6 | 1 | 16.7% | 2 | 0.333 | 2.0 |
| Serranidae | Teleost | *Epinephelus adscensionis* | 3 | 1.2% | 3 | 0.012 | 1.0 | 6 | 4.2% | 7 | 0.049 | 1.2 | 0 | 0.0% | 0 | 0.000 | NA |
| Serranidae | Teleost | *Epinephelus aeneus* | 1 | 0.4% | 1 | 0.004 | 1.0 | 1 | 0.7% | 1 | 0.007 | 1.0 | 0 | 0.0% | 0 | 0.000 | NA |
| Serranidae | Teleost | *Epinephelus costae* | 0 | 0.0% | 0 | 0.000 | NA | 1 | 0.7% | 2 | 0.014 | 2.0 | 0 | 0.0% | 0 | 0.000 | NA |

**S3 Table (cont.)**

| **Family** | **Type** | **Species** | **Príncipe** | | | | | **São Tomé** | | | | | **Tinhosas** | | | | |
| --- | --- | --- | --- | --- | --- | --- | --- | --- | --- | --- | --- | --- | --- | --- | --- | --- | --- |
|  |  |  | **Occ.** | **Occ. (% depl.)** | **MaxN total** | **Mean MaxN per depl.** | **Mean MaxN per occ.** | **Occ.** | **Occ. (% depl.)** | **MaxN total** | **Mean MaxN per depl.** | **Mean MaxN per occ.** | **Occ.** | **Occ. (% depl.)** | **MaxN total** | **Mean MaxN per depl.** | **Mean MaxN per occ.** |
| Serranidae | Teleost | *Paranthias furcifer* | 53 | 20.4% | 2702 | 10.392 | 51.0 | 56 | 38.9% | 3757 | 26.090 | 67.1 | 6 | 100.0% | 745 | 124.167 | 124.2 |
| Serranidae | Teleost | *Rypticus saponaceus* | 34 | 13.1% | 36 | 0.138 | 1.1 | 31 | 21.5% | 37 | 0.257 | 1.2 | 3 | 50.0% | 3 | 0.500 | 1.0 |
| Serranidae | Teleost | *Serranus accraensis* | 1 | 0.4% | 2 | 0.008 | 2.0 | 0 | 0.0% | 0 | 0.000 | NA | 0 | 0.0% | 0 | 0.000 | NA |
| Serranidae | Teleost | *Serranus cabrilla* | 18 | 6.9% | 28 | 0.108 | 1.6 | 7 | 4.9% | 25 | 0.174 | 3.6 | 0 | 0.0% | 0 | 0.000 | NA |
| Serranidae | Teleost | *Serranus pulcher* | 105 | 40.4% | 661 | 2.542 | 6.3 | 37 | 25.7% | 136 | 0.944 | 3.7 | 1 | 16.7% | 1 | 0.167 | 1.0 |
| Sparidae | Teleost | *Boops boops* | 0 | 0.0% | 0 | 0.000 | NA | 2 | 1.4% | 11 | 0.076 | 5.5 | 0 | 0.0% | 0 | 0.000 | NA |
| Sparidae | Teleost | *Pagrus caeruleostictus* | 8 | 3.1% | 16 | 0.062 | 2.0 | 20 | 13.9% | 72 | 0.500 | 3.6 | 0 | 0.0% | 0 | 0.000 | NA |
| Sparidae | Teleost | *Pagrus pagrus* | 1 | 0.4% | 1 | 0.004 | 1.0 | 0 | 0.0% | 0 | 0.000 | NA | 0 | 0.0% | 0 | 0.000 | NA |
| Sparidae | Teleost | *Spicara melanurus* | 4 | 1.5% | 97 | 0.373 | 24.3 | 4 | 2.8% | 94 | 0.653 | 23.5 | 1 | 16.7% | 31 | 5.167 | 31.0 |
| Sparidae | Teleost | *Spicara nigricauda* | 3 | 1.2% | 18 | 0.069 | 6.0 | 0 | 0.0% | 0 | 0.000 | NA | 0 | 0.0% | 0 | 0.000 | NA |
| Sphyraenidae | Teleost | *Sphyraena barracuda* | 32 | 12.3% | 35 | 0.135 | 1.1 | 5 | 3.5% | 5 | 0.035 | 1.0 | 5 | 83.3% | 5 | 0.833 | 1.0 |
| Syngnathidae | Teleost | *Hippocampus algiricus* | 1 | 0.4% | 1 | 0.004 | 1.0 | 0 | 0.0% | 0 | 0.000 | NA | 0 | 0.0% | 0 | 0.000 | NA |
| Syngnathidae | Teleost | *Microphis aculeatus* | 4 | 1.5% | 4 | 0.015 | 1.0 | 0 | 0.0% | 0 | 0.000 | NA | 0 | 0.0% | 0 | 0.000 | NA |
| Tetraodontidae | Teleost | *Canthigaster supramacula* | 35 | 13.5% | 56 | 0.215 | 1.6 | 32 | 22.2% | 62 | 0.431 | 1.9 | 4 | 66.7% | 6 | 1.000 | 1.5 |
| Tetraodontidae | Teleost | *Lagocephalus laevigatus* | 6 | 2.3% | 10 | 0.038 | 1.7 | 10 | 6.9% | 51 | 0.354 | 5.1 | 0 | 0.0% | 0 | 0.000 | NA |
| Tetraodontidae | Teleost | *Sphoeroides marmoratus* | 42 | 16.2% | 57 | 0.219 | 1.4 | 16 | 11.1% | 22 | 0.153 | 1.4 | 0 | 0.0% | 0 | 0.000 | NA |
| Trachinidae | Teleost | *Trachinus lineolatus* | 1 | 0.4% | 1 | 0.004 | 1.0 | 0 | 0.0% | 0 | 0.000 | NA | 0 | 0.0% | 0 | 0.000 | NA |
| Uranoscopidae | Teleost | *Uranoscopus polli* | 0 | 0.0% | 0 | 0.000 | NA | 1 | 0.7% | 1 | 0.007 | 1.0 | 0 | 0.0% | 0 | 0.000 | NA |
