# Supplementary material for: Fish community composition in the tropical archipelago of São Tomé and Príncipe
Source: PLoS One. 2024 Nov 1;19(11):e0312849. doi: 10.1371/journal.pone.0312849 (PMC11530061; doi:10.1371/journal.pone.0312849)
Supplement: S4 Table — (DOCX) [file pone.0312849.s010.docx]

**S4 Table**: Family’s species richness, occurrence (defined as number of deployments and percentage of deployments in which a species is observed), and abundance (defined as MaxN totals and mean MaxN per deployment)

| **Family** | **Type** | **Príncipe** | | | | | **São Tomé** | | | | | **Tinhosas** | | | | |
| --- | --- | --- | --- | --- | --- | --- | --- | --- | --- | --- | --- | --- | --- | --- | --- | --- |
|  |  | **Richn.** | **Occ. (n depl.)** | **Occ. (% depl.)** | **MaxN totals** | **Mean MaxN per depl.** | **Richn.** | **Occ. (n depl.)** | **Occ. (% depl.)** | **MaxN totals** | **Mean MaxN per depl.** | **Richn.** | **Occ. (n depl.)** | **Occ. (% depl.)** | **MaxN totals** | **Mean MaxN per depl.** |
| Elasmobranch | Carcharhinidae | 3 | 4 | 1.5% | 8 | 0.031 | 1 | 1 | 0.7% | 1 | 0.007 | 0 | 0 | 0.0% | 0 | 0.000 |
| Elasmobranch | Dasyatidae | 4 | 18 | 6.9% | 21 | 0.081 | 1 | 4 | 2.8% | 4 | 0.028 | 0 | 0 | 0.0% | 0 | 0.000 |
| Elasmobranch | Ginglymostomatidae | 1 | 3 | 1.2% | 4 | 0.015 | 0 | 0 | 0.0% | 0 | 0.000 | 0 | 0 | 0.0% | 0 | 0.000 |
| Elasmobranch | Mobulidae | 1 | 1 | 0.4% | 1 | 0.004 | 1 | 1 | 0.7% | 1 | 0.007 | 0 | 0 | 0.0% | 0 | 0.000 |
| Elasmobranch | Sphyrnidae | 0 | 0 | 0.0% | 0 | 0.000 | 1 | 5 | 3.5% | 5 | 0.035 | 0 | 0 | 0.0% | 0 | 0.000 |
| Teleost | Acanthuridae | 2 | 60 | 23.1% | 908 | 3.492 | 2 | 48 | 33.3% | 222 | 1.542 | 2 | 4 | 66.7% | 18 | 3.000 |
| Teleost | Apogonidae | 1 | 1 | 0.4% | 1 | 0.004 | 0 | 0 | 0.0% | 0 | 0.000 | 0 | 0 | 0.0% | 0 | 0.000 |
| Teleost | Atherinidae | 1 | 1 | 0.4% | 2 | 0.008 | 0 | 0 | 0.0% | 0 | 0.000 | 0 | 0 | 0.0% | 0 | 0.000 |
| Teleost | Aulostomidae | 1 | 4 | 1.5% | 4 | 0.015 | 1 | 23 | 16.0% | 38 | 0.264 | 1 | 3 | 50.0% | 8 | 1.333 |
| Teleost | Balistidae | 3 | 91 | 35.0% | 160 | 0.615 | 4 | 58 | 40.3% | 90 | 0.625 | 3 | 5 | 83.3% | 37 | 6.167 |
| Teleost | Belonidae | 0 | 0 | 0.0% | 0 | 0.000 | 1 | 1 | 0.7% | 1 | 0.007 | 0 | 0 | 0.0% | 0 | 0.000 |
| Teleost | Blenniidae | 2 | 9 | 3.5% | 11 | 0.042 | 2 | 12 | 8.3% | 23 | 0.160 | 1 | 2 | 33.3% | 3 | 0.500 |
| Teleost | Bothidae | 2 | 54 | 20.8% | 73 | 0.281 | 1 | 34 | 23.6% | 44 | 0.306 | 0 | 0 | 0.0% | 0 | 0.000 |
| Teleost | Carangidae | 13 | 223 | 85.8% | 2475 | 9.519 | 10 | 72 | 50.0% | 625 | 4.340 | 3 | 6 | 100.0% | 28 | 4.667 |
| Teleost | Chaetodontidae | 1 | 4 | 1.5% | 6 | 0.023 | 1 | 21 | 14.6% | 37 | 0.257 | 0 | 0 | 0.0% | 0 | 0.000 |
| Teleost | Cirrhitidae | 1 | 26 | 10.0% | 32 | 0.123 | 1 | 17 | 11.8% | 25 | 0.174 | 1 | 4 | 66.7% | 5 | 0.833 |
| Teleost | Clupeidae | 1 | 2 | 0.8% | 24 | 0.092 | 0 | 1 | 0.7% | 15 | 0.104 | 0 | 0 | 0.0% | 0 | 0.000 |
| Teleost | Congridae | 3 | 13 | 5.0% | 133 | 0.512 | 1 | 1 | 0.7% | 47 | 0.326 | 0 | 0 | 0.0% | 0 | 0.000 |
| Teleost | Dactylopteridae | 1 | 60 | 23.1% | 84 | 0.323 | 1 | 33 | 22.9% | 60 | 0.417 | 0 | 0 | 0.0% | 0 | 0.000 |
| Teleost | Diodontidae | 4 | 22 | 8.5% | 23 | 0.088 | 3 | 14 | 9.7% | 14 | 0.097 | 0 | 0 | 0.0% | 0 | 0.000 |
| Teleost | Echeneidae | 1 | 9 | 3.5% | 18 | 0.069 | 1 | 7 | 4.9% | 8 | 0.056 | 1 | 1 | 16.7% | 1 | 0.167 |
| Teleost | Ephippidae | 0 | 0 | 0.0% | 0 | 0.000 | 1 | 1 | 0.7% | 1 | 0.007 | 0 | 0 | 0.0% | 0 | 0.000 |
| Teleost | Fistulariidae | 1 | 3 | 1.2% | 3 | 0.012 | 1 | 21 | 14.6% | 29 | 0.201 | 0 | 0 | 0.0% | 0 | 0.000 |
| Teleost | Gerreidae | 0 | 0 | 0.0% | 0 | 0.000 | 1 | 1 | 0.7% | 2 | 0.014 | 0 | 0 | 0.0% | 0 | 0.000 |
| Teleost | Gobiidae | 2 | 26 | 10.0% | 51 | 0.196 | 1 | 10 | 6.9% | 13 | 0.090 | 0 | 0 | 0.0% | 0 | 0.000 |
| Teleost | Haemulidae | 0 | 0 | 0.0% | 0 | 0.000 | 1 | 2 | 1.4% | 21 | 0.146 | 1 | 1 | 16.7% | 1 | 0.167 |
| Teleost | Holocentridae | 2 | 23 | 8.8% | 121 | 0.465 | 3 | 20 | 13.9% | 82 | 0.569 | 2 | 3 | 50.0% | 19 | 3.167 |
| Teleost | Kyphosidae | 2 | 16 | 6.2% | 100 | 0.385 | 1 | 2 | 1.4% | 3 | 0.021 | 0 | 3 | 50.0% | 9 | 1.500 |
| Teleost | Labridae | 7 | 134 | 51.5% | 625 | 2.404 | 6 | 75 | 52.1% | 605 | 4.201 | 5 | 6 | 100.0% | 75 | 12.500 |
| Teleost | Labrisomidae | 1 | 1 | 0.4% | 1 | 0.004 | 1 | 4 | 2.8% | 7 | 0.049 | 0 | 0 | 0.0% | 0 | 0.000 |
| Teleost | Lethrinidae | 1 | 60 | 23.1% | 287 | 1.104 | 1 | 29 | 20.1% | 65 | 0.451 | 0 | 0 | 0.0% | 0 | 0.000 |
| Teleost | Lutjanidae | 7 | 58 | 22.3% | 247 | 0.950 | 6 | 29 | 20.1% | 122 | 0.847 | 3 | 5 | 83.3% | 67 | 11.167 |
| Teleost | Megalopidae | 0 | 0 | 0.0% | 0 | 0.000 | 1 | 1 | 0.7% | 1 | 0.007 | 0 | 0 | 0.0% | 0 | 0.000 |
| Teleost | Monacanthidae | 5 | 78 | 30.0% | 135 | 0.519 | 5 | 85 | 59.0% | 530 | 3.681 | 2 | 6 | 100.0% | 15 | 2.500 |
| Teleost | Mullidae | 3 | 36 | 13.8% | 235 | 0.904 | 2 | 57 | 39.6% | 329 | 2.285 | 1 | 6 | 100.0% | 39 | 6.500 |
| Teleost | Muraenidae | 6 | 25 | 9.6% | 42 | 0.162 | 6 | 27 | 18.8% | 49 | 0.340 | 1 | 1 | 16.7% | 2 | 0.333 |
| Teleost | Ophichthidae | 2 | 7 | 2.7% | 7 | 0.027 | 4 | 6 | 4.2% | 7 | 0.049 | 0 | 0 | 0.0% | 0 | 0.000 |
| Teleost | Ostraciidae | 2 | 42 | 16.2% | 60 | 0.231 | 1 | 41 | 28.5% | 50 | 0.347 | 0 | 0 | 0.0% | 0 | 0.000 |
| Teleost | Pomacanthidae | 1 | 39 | 15.0% | 67 | 0.258 | 1 | 20 | 13.9% | 32 | 0.222 | 1 | 5 | 83.3% | 10 | 1.667 |
| Teleost | Pomacentridae | 6 | 45 | 17.3% | 1064 | 4.092 | 5 | 44 | 30.6% | 1447 | 10.049 | 4 | 5 | 83.3% | 423 | 70.500 |
| Teleost | Priacanthidae | 1 | 1 | 0.4% | 1 | 0.004 | 1 | 2 | 1.4% | 2 | 0.014 | 1 | 1 | 16.7% | 3 | 0.500 |
| Teleost | Scaridae | 3 | 55 | 21.2% | 265 | 1.019 | 3 | 58 | 40.3% | 237 | 1.646 | 4 | 6 | 100.0% | 29 | 4.833 |
| Teleost | Scombridae | 6 | 15 | 5.8% | 17 | 0.065 | 2 | 18 | 12.5% | 27 | 0.188 | 0 | 0 | 0.0% | 0 | 0.000 |

**S4 Table (cont.)**

| **Family** | **Type** | **Príncipe** | | | | | **São Tomé** | | | | | **Tinhosas** | | | | |
| --- | --- | --- | --- | --- | --- | --- | --- | --- | --- | --- | --- | --- | --- | --- | --- | --- |
|  |  | **Richn.** | **Occ. (n depl.)** | **Occ. (% depl.)** | **MaxN totals** | **Mean MaxN per depl.** | **Richn.** | **Occ. (n depl.)** | **Occ. (% depl.)** | **MaxN totals** | **Mean MaxN per depl.** | **Richn.** | **Occ. (n depl.)** | **Occ. (% depl.)** | **MaxN totals** | **Mean MaxN per depl.** |
| Teleost | Scorpaenidea | 1 | 1 | 0.4% | 1 | 0.004 | 1 | 1 | 0.7% | 1 | 0.007 | 0 | 0 | 0.0% | 0 | 0.000 |
| Teleost | Serranidae | 9 | 171 | 65.8% | 3582 | 13.777 | 9 | 85 | 59.0% | 4127 | 28.660 | 4 | 6 | 100.0% | 751 | 125.167 |
| Teleost | Sparidae | 4 | 17 | 6.5% | 162 | 0.623 | 3 | 26 | 18.1% | 177 | 1.229 | 1 | 1 | 16.7% | 31 | 5.167 |
| Teleost | Sphyraenidae | 1 | 32 | 12.3% | 35 | 0.135 | 1 | 5 | 3.5% | 5 | 0.035 | 1 | 5 | 83.3% | 5 | 0.833 |
| Teleost | Syngnathidae | 2 | 5 | 1.9% | 5 | 0.019 | 0 | 0 | 0.0% | 0 | 0.000 | 0 | 0 | 0.0% | 0 | 0.000 |
| Teleost | Tetraodontidae | 3 | 81 | 31.2% | 123 | 0.473 | 3 | 57 | 39.6% | 135 | 0.938 | 1 | 4 | 66.7% | 6 | 1.000 |
| Teleost | Trachinidae | 1 | 1 | 0.4% | 1 | 0.004 | 0 | 0 | 0.0% | 0 | 0.000 | 0 | 0 | 0.0% | 0 | 0.000 |
| Teleost | Uranoscopidae | 0 | 0 | 0.0% | 0 | 0.000 | 1 | 1 | 0.7% | 1 | 0.007 | 0 | 0 | 0.0% | 0 | 0.000 |
