# Supplementary material for: Fish community composition in the tropical archipelago of São Tomé and Príncipe
Source: PLoS One. 2024 Nov 1;19(11):e0312849. doi: 10.1371/journal.pone.0312849 (PMC11530061; doi:10.1371/journal.pone.0312849)
Supplement: S5 Table — (DOCX) [file pone.0312849.s011.docx]

**S5 Table**: Mean and standard deviation of species richness (S), relative abundance (MaxN) and E_1/D_ evenness Index, disaggregated by habitat and island.

| **Variable** | **Island** | **All habitats** | | | **Sand** | | | **Maerl** | | | **Rock** | | |
| --- | --- | --- | --- | --- | --- | --- | --- | --- | --- | --- | --- | --- | --- |
|  |  | **Mean** | **SD** | **n** | **Mean** | **SD** | **n** | **Mean** | **SD** | **n** | **Mean** | **SD** | **n** |
| **Richness (S)** | **ST & P** | 8.9 | 7.6 | 417 | 8.2 | 4.6 | 120 | 20.2 | 5.8 | 93 | 4.1 | 2.8 | 204 |
|  | **São Tomé** | 9.8 | 8.0 | 148 | 11.0 | 5.0 | 41 | 20.4 | 5.2 | 36 | 3.7 | 3.0 | 71 |
|  | **Príncipe** | 8.1 | 7.0 | 263 | 6.7 | 3.7 | 79 | 19.7 | 6.3 | 51 | 4.4 | 2.7 | 133 |
| **Relative abundance (MaxN)** | **ST & P** | 53.2 | 87.4 | 417 | 47.6 | 58.5 | 120 | 146.3 | 126.4 | 93 | 14.0 | 29.3 | 204 |
|  | **São Tomé** | 63.3 | 98.8 | 148 | 72.9 | 60.6 | 41 | 150.4 | 150.8 | 36 | 13.6 | 24.2 | 71 |
|  | **Príncipe** | 42.7 | 69.1 | 263 | 34.5 | 53.2 | 79 | 129.5 | 89.4 | 51 | 14.3 | 31.8 | 133 |
| **E_1/D_ evenness index** | **ST & P** | 0.56 | 0.30 | 384 | 0.51 | 0.25 | 120 | 0.26 | 0.17 | 92 | 0.75 | 0.23 | 172 |
|  | **São Tomé** | 0.50 | 0.31 | 132 | 0.41 | 0.27 | 41 | 0.24 | 0.11 | 36 | 0.73 | 0.26 | 55 |
|  | **Príncipe** | 0.60 | 0.28 | 246 | 0.55 | 0.22 | 79 | 0.29 | 0.21 | 50 | 0.76 | 0.21 | 117 |
