# Supplementary material for: Fish community composition in the tropical archipelago of São Tomé and Príncipe
Source: PLoS One. 2024 Nov 1;19(11):e0312849. doi: 10.1371/journal.pone.0312849 (PMC11530061; doi:10.1371/journal.pone.0312849)
Supplement: S6 Table — Habitat and island were fitted as categorical effects; depth, distance to shore and slope were fitted as thin plate splines; and season and season by habitat were fitted as cyclic splines. Variable smooths were fitted via Restricted Maximum Likelihood (REML). (DOCX) [file pone.0312849.s012.docx]

**S6** **Table** Generalised Additive Models (GAMs) to assess the effect of environmental variables (depth, distance to shore, Slope, Habitat, Island and Season) on Richness (S), Abundance (MaxN), and E1/D Evenness Index. Habitat and island were fitted as categorical effects; depth, distance to shore and slope were fitted as thin plate splines; and season and season by habitat were fitted as cyclic splines. Variable smooths were fitted via Restricted Maximum Likelihood (REML).

| **Response** | **Intercept** | **Predictors** | | | | | | | **R^2^** | **df** | **logLik** | **AIC** | **ΔAIC** | **Model weight** |
| --- | --- | --- | --- | --- | --- | --- | --- | --- | --- | --- | --- | --- | --- | --- |
|  |  | **s(Depth)** | **s(Dist. to shore)** | **Habitat** | **Island** | **s(Season)** | **s(Season, by = island)** | **s(Slope)** |  |  |  |  |  |  |
| **Richness** | 2.03 | + |  | + |  |  | + | + | 0.65 | 9 | -1081.9 | 2182.6 | 0.00 | 0.37 |
| **Richness** | 2.02 | + |  | + |  | + |  | + | 0.65 | 11 | -1080.3 | 2182.6 | 0.01 | 0.36 |
| **Richness** | 2.01 | + |  | + | + | + | + |  | 0.65 | 9 | -1083.2 | 2186.3 | 3.71 | 0.06 |
| **Richness** | 2.01 | + |  | + | + |  | + |  | 0.65 | 9 | -1083.2 | 2186.3 | 3.71 | 0.06 |
| **Richness** | 2.04 | + |  | + |  | + | + |  | 0.65 | 9 | -1084.2 | 2186.4 | 3.82 | 0.05 |
| **Richness** | 2.04 | + |  | + |  |  | + |  | 0.65 | 9 | -1084.2 | 2186.4 | 3.82 | 0.05 |
| **Richness** | 2.03 | + |  | + |  | + |  |  | 0.65 | 10 | -1082.8 | 2186.8 | 4.18 | 0.05 |
| **Abundance** | 3.65 | + | + | + | + |  | + |  | 0.51 | 13 | -1835.5 | 3697.1 | 0.00 | 0.22 |
| **Abundance** | 3.75 | + | + | + |  |  | + |  | 0.50 | 11 | -1837.2 | 3697.9 | 0.72 | 0.15 |
| **Abundance** | 3.67 | + |  | + | + | + | + | + | 0.50 | 9 | -1839.9 | 3698.4 | 1.25 | 0.11 |
| **Abundance** | 3.67 | + |  | + | + |  | + | + | 0.50 | 9 | -1839.9 | 3698.4 | 1.28 | 0.11 |
| **Abundance** | 3.76 | + |  | + |  | + |  | + | 0.49 | 6 | -1842.7 | 3698.8 | 1.63 | 0.10 |
| **Abundance** | 3.76 | + |  | + |  |  |  | + | 0.49 | 6 | -1842.7 | 3698.8 | 1.64 | 0.09 |
| **Abundance** | 3.78 | + | + | + |  |  |  |  | 0.49 | 9 | -1840.5 | 3699.3 | 2.16 | 0.07 |
| **Abundance** | 3.68 |  |  | + | + | + | + | + | 0.49 | 8 | -1841.8 | 3700.7 | 3.54 | 0.04 |
| **Abundance** | 3.68 |  |  | + | + |  | + | + | 0.49 | 8 | -1841.8 | 3700.7 | 3.57 | 0.04 |
| **Abundance** | 3.64 |  | + | + | + |  | + |  | 0.50 | 12 | -1838.1 | 3700.8 | 3.69 | 0.03 |
| **Abundance** | 3.67 | + |  | + | + |  | + |  | 0.49 | 8 | -1843.2 | 3702.8 | 5.63 | 0.01 |
| **Abundance** | 3.71 | + |  | + | + | + |  |  | 0.48 | 6 | -1845.4 | 3702.8 | 5.68 | 0.01 |
| **Abundance** | 3.71 | + |  | + | + |  |  |  | 0.48 | 6 | -1845.3 | 3702.9 | 5.77 | 0.01 |
| **Evenness** | -2.52 | + |  | + |  |  | + | + | 0.40 | 9 | 582.1 | -1145.5 | 0.00 | 0.29 |
| **Evenness** | -2.53 | + |  | + |  | + | + |  | 0.39 | 8 | 580.2 | -1143.7 | 1.76 | 0.12 |
| **Evenness** | -2.53 | + |  | + |  |  | + |  | 0.39 | 8 | 580.2 | -1143.7 | 1.76 | 0.12 |
| **Evenness** | -2.54 |  |  | + |  | + | + | + | 0.39 | 8 | 580.2 | -1143.5 | 1.96 | 0.11 |
| **Evenness** | -2.54 |  |  | + |  |  | + | + | 0.39 | 8 | 580.2 | -1143.5 | 1.96 | 0.11 |
| **Evenness** | -2.52 | + |  | + |  | + |  | + | 0.38 | 6 | 577.3 | -1142.6 | 2.90 | 0.07 |
| **Evenness** | -2.52 | + |  | + |  |  |  | + | 0.38 | 6 | 577.3 | -1142.6 | 2.90 | 0.07 |
| **Evenness** | -2.55 |  |  | + |  |  | + |  | 0.39 | 7 | 578.2 | -1141.4 | 4.08 | 0.04 |
| **Evenness** | -2.53 | + |  | + |  | + |  |  | 0.38 | 5 | 575.6 | -1141.2 | 4.30 | 0.03 |
| **Evenness** | -2.53 | + |  | + |  |  |  |  | 0.38 | 5 | 575.6 | -1141.2 | 4.30 | 0.03 |
| **Evenness** | -2.54 |  |  | + |  |  |  | + | 0.38 | 5 | 575.0 | -1140.0 | 5.45 | 0.02 |
