# Supplementary material for: Fish community composition in the tropical archipelago of São Tomé and Príncipe
Source: PLoS One. 2024 Nov 1;19(11):e0312849. doi: 10.1371/journal.pone.0312849 (PMC11530061; doi:10.1371/journal.pone.0312849)
Supplement: S7 Table — Values closer to 1 indicate stronger and more consistent support for a predictor across the retained models in the top model set. (DOCX) [file pone.0312849.s013.docx]

**S7 Table**: Degree of support of the effect of environmental variables on diversity indicators, expressed as the summed weights of models in S6 Table. Values closer to 1 indicate stronger and more consistent support for a predictor across the retained models in the top model set.

| **Variable** | **Level** | **Richness (*S’*)** | | | **Relative abundance (MaxN)** | | | **Evenness (E_1/D_)** | | | |
| --- | --- | --- | --- | --- | --- | --- | --- | --- | --- | --- | --- |
|  |  | **Estimate** | **Std. error** | **Summed Weights** | **Estimate** | **Std. error** | **Summed Weights** | **Estimate** | **Std. error** | **Summed Weights** |  |
| **(Intercept)** | | 2.02 | 0.05 | NA | 3.70 | 0.11 | NA | -2.53 | 0.08 | NA |  |
| **Depth** | | NA | NA | 1.00 | NA | NA | 0.89 | NA | NA | 0.73 |  |
| **Slope** | | NA | NA | 0.73 | NA | NA | 0.49 | NA | NA | 0.66 |  |
| **Dist. to shore** | | NA | NA | 0.00 | NA | NA | 0.47 | NA | NA | 0.00 |  |
| **Habitat** | **Habitat: Rock** | 1.04 | 0.07 | 1.00 | 1.16 | 0.17 | 1.00 | -0.74 | 0.15 | 1.00 |  |
|  | **Habitat: Sand** | -0.61 | 0.06 |  | -1.10 | 0.12 |  | 0.76 | 0.09 |  |  |
| **Island** | **Island: São Tomé** | 0.01 | 0.03 | 0.11 | 0.11 | 0.13 | 0.59 | 0.00 | 0.00 | 0.00 |  |
| **s(Season)** | | NA | NA | 0.52 | NA | NA | 0.26 | NA | NA | 0.33 |  |
| **s(Season, by = island)** | | NA | NA | 0.59 | NA | NA | 0.71 | NA | NA | 0.78 |  |
