# Supplementary material for: Fish community composition in the tropical archipelago of São Tomé and Príncipe
Source: PLoS One. 2024 Nov 1;19(11):e0312849. doi: 10.1371/journal.pone.0312849 (PMC11530061; doi:10.1371/journal.pone.0312849)
Supplement: S8 Table — Grey-shaded cells indicate that the variable was not included in the model. (DOCX) [file pone.0312849.s014.docx]

**S8** **Table**: Contribution of predictors to the deviance explained by each model in the top model set. Grey-shaded cells indicate that the variable was not included in the model.

| **Response** | **Contribution of predictors to explained deviance** | | | | | | | **Deviance explained** | **Model weight** |
| --- | --- | --- | --- | --- | --- | --- | --- | --- | --- |
|  | **s(Depth)** | **s(Dist. to shore)** | **Habitat** | **Island** | **s(Season)** | **s(Season, by = island)** | **s(Slope)** |  |  |
| **Richness** | 6.4% |  | 78.1% |  |  | 7.0% | 8.5% | 65.6% | 0.37 |
| **Richness** | 6.1% |  | 78.2% |  | 7.1% |  | 8.6% | 65.9% | 0.36 |
| **Richness** | 7.6% |  | 83.8% | 0.7% | 3.9% | 4.1% |  | 65.4% | 0.06 |
| **Richness** | 7.7% |  | 84.8% | 0.9% |  | 6.6% |  | 65.4% | 0.06 |
| **Richness** | 7.5% |  | 84.0% |  | 4.2% | 4.3% |  | 65.2% | 0.05 |
| **Richness** | 7.6% |  | 85.3% |  |  | 7.2% |  | 65.2% | 0.05 |
| **Richness** | 7.4% |  | 85.5% |  | 7.1% |  |  | 65.4% | 0.05 |
| **Abundance** | 3.3% | 17.8% | 66.9% | 1.9% |  | 10.1% |  | 45.8% | 0.22 |
| **Abundance** | 3.0% | 18.4% | 67.7% |  |  | 11.0% |  | 45.4% | 0.15 |
| **Abundance** | 3.5% |  | 71.7% | 1.5% | 4.6% | 7.0% | 11.6% | 44.8% | 0.11 |
| **Abundance** | 3.5% |  | 74.0% | 1.7% |  | 9.2% | 11.7% | 44.8% | 0.11 |
| **Abundance** | 3.6% |  | 76.7% |  | 7.4% |  | 12.4% | 44.1% | 0.10 |
| **Abundance** | 3.3% |  | 83.9% |  |  |  | 12.8% | 44.1% | 0.09 |
| **Abundance** | 3.5% | 17.7% | 78.9% |  |  |  |  | 44.6% | 0.07 |
| **Abundance** |  |  | 73.9% | 1.4% | 4.7% | 7.2% | 12.8% | 44.3% | 0.04 |
| **Abundance** |  |  | 76.2% | 1.5% |  | 9.5% | 12.8% | 44.3% | 0.04 |
| **Abundance** |  | 19.2% | 68.9% | 1.5% |  | 10.5% |  | 45.2% | 0.03 |
| **Abundance** | 4.6% |  | 83.7% | 2.4% |  | 9.3% |  | 44.0% | 0.01 |
| **Abundance** | 4.9% |  | 85.6% | 2.5% | 7.0% |  |  | 43.4% | 0.01 |
| **Abundance** | 4.9% |  | 91.7% | 3.4% |  |  |  | 43.4% | 0.01 |
| **Evenness** | 6.1% |  | 75.0% |  |  | 9.8% | 9.1% | 47.2% | 0.29 |
| **Evenness** | 7.3% |  | 80.1% |  | 4.5% | 8.2% |  | 46.5% | 0.12 |
| **Evenness** | 7.2% |  | 82.4% |  |  | 10.4% |  | 46.5% | 0.12 |
| **Evenness** |  |  | 77.9% |  | 4.3% | 7.7% | 10.1% | 46.4% | 0.11 |
| **Evenness** |  |  | 80.0% |  |  | 9.8% | 10.2% | 46.4% | 0.11 |
| **Evenness** | 6.2% |  | 77.6% |  | 6.6% |  | 9.6% | 45.4% | 0.07 |
| **Evenness** | 6.1% |  | 84.1% |  |  |  | 9.9% | 45.4% | 0.07 |
| **Evenness** |  |  | 89.6% |  |  | 10.5% |  | 45.7% | 0.04 |
| **Evenness** | 7.5% |  | 85.5% |  | 7.0% |  |  | 44.7% | 0.03 |
| **Evenness** | 7.3% |  | 92.7% |  |  |  |  | 44.7% | 0.03 |
| **Evenness** |  |  | 88.8% |  |  |  | 11.2% | 44.5% | 0.02 |
