# Supplementary material for: Fish community composition in the tropical archipelago of São Tomé and Príncipe
Source: PLoS One. 2024 Nov 1;19(11):e0312849. doi: 10.1371/journal.pone.0312849 (PMC11530061; doi:10.1371/journal.pone.0312849)
Supplement: S9 Table — (DOCX) [file pone.0312849.s015.docx]

**S9 Table**: Results of an ANOVA-like permutation significance test for the effect of each dbRDA constrained axis on response variables, conducted with the function vegan::anova.cca() in R, and percentage of constrained variance explained by each of the constrained axes.

|  | **Df** | **SumOfSqs** | **F** | **Pr(>F)** | **% of constrained variance** |
| --- | --- | --- | --- | --- | --- |
| **dbRDA1** | 1 | 25.04 | 90.94 | 0.001 | 61.6% |
| **dbRDA2** | 1 | 8.39 | 30.49 | 0.001 | 20.7% |
| **dbRDA3** | 1 | 4.02 | 14.61 | 0.001 | 9.9% |
| **dbRDA4** | 1 | 1.33 | 4.84 | 0.002 | 3.3% |
| **dbRDA5** | 1 | 0.73 | 2.65 | 0.046 | 1.8% |
| **dbRDA6** | 1 | 0.48 | 1.74 | 0.332 | 1.2% |
| **dbRDA7** | 1 | 0.37 | 1.35 | 0.458 | 0.9% |
| **dbRDA8** | 1 | 0.25 | 0.89 | 0.557 | 0.6% |
| **Residual** | 398 | 109.57 |  |  |  |
