# Supplementary material for: Fish community composition in the tropical archipelago of São Tomé and Príncipe
Source: PLoS One. 2024 Nov 1;19(11):e0312849. doi: 10.1371/journal.pone.0312849 (PMC11530061; doi:10.1371/journal.pone.0312849)
Supplement: S10 Table — Constrained dbRDA axes are orthogonal combinations of the explanatory variables (i.e., a multiple regression model) that best explain, in successive order, the variation of the response matrix (Borcard, Gillet & Legendre, 2011). (DOCX) [file pone.0312849.s016.docx]

**S10 Table**: Variable coefficients for each of the constrained dbRDA axes and centroids of factor variables on each of the constrained axes. Constrained dbRDA axes are orthogonal combinations of the explanatory variables (i.e., a multiple regression model) that best explain, in successive order, the variation of the response matrix (Borcard, Gillet & Legendre, 2011).

|  | **dbRDA1** | **dbRDA2** | **dbRDA3** | **dbRDA4** | **dbRDA5** | **dbRDA6** |
| --- | --- | --- | --- | --- | --- | --- |
| **COEFFICIENTS** | | | | | | |
| **Habitat: Rock^1^** | 0.94 | -0.05 | 0.27 | -0.15 | 0.03 | -0.10 |
| **Habitat: Sand^1^** | -0.60 | -0.80 | 0.03 | -0.01 | 0.01 | 0.02 |
| **Island: São Tomé^2^** | 0.31 | -0.25 | -0.84 | 0.10 | -0.34 | 0.06 |
| **Island: Tinhosas^2^** | 0.20 | 0.01 | 0.14 | -0.18 | 0.16 | 0.69 |
| **Slope** | 0.40 | 0.10 | 0.12 | 0.09 | -0.18 | 0.40 |
| **Dist. to shore** | -0.42 | 0.31 | -0.21 | -0.48 | 0.33 | -0.49 |
| **Depth** | -0.47 | 0.18 | -0.29 | -0.71 | -0.26 | 0.22 |
| **Season^3^** | -0.30 | 0.06 | 0.35 | 0.09 | -0.70 | -0.28 |
| **CENTROIDS** | | | | | | |
| **Habitat: Maerl** | -0.25 | 1.11 | -0.35 | 0.18 | -0.05 | 0.09 |
| **Habitat: Rock** | 1.34 | -0.07 | 0.39 | -0.22 | 0.05 | -0.14 |
| **Habitat: Sand** | -0.49 | -0.65 | 0.03 | 0.00 | 0.01 | 0.01 |
| **Island: Príncipe** | -0.21 | 0.14 | 0.47 | -0.03 | 0.18 | -0.14 |
| **Island: São Tomé** | 0.33 | -0.26 | -0.89 | 0.11 | -0.36 | 0.07 |
| **Island: Tinhosas** | 1.26 | 0.06 | 0.92 | -1.17 | 1.00 | 4.42 |

**TABLE NOTES**: **(1)** Reference level: Maerl; **(2)** Reference level: Príncipe
