# Supplementary material for: Fish community composition in the tropical archipelago of São Tomé and Príncipe
Source: PLoS One. 2024 Nov 1;19(11):e0312849. doi: 10.1371/journal.pone.0312849 (PMC11530061; doi:10.1371/journal.pone.0312849)
Supplement: S11 Table — Significance of the contribution of each species to overall dissimilarities between pairs was assessed using permutation tests. (DOCX) [file pone.0312849.s017.docx]

**S11 Table**: SIMPER pairwise comparisons of habitat types, with mean MaxN (and SD), and average contribution of each species to overall dissimilarities amongst pairs. Significance of the contribution of each species to overall dissimilarities between pairs was assessed using permutation tests.

| **Species** | **Mean MaxN (SD)** | | | **Average contribution to dissimilarities and significance** | | |
| --- | --- | --- | --- | --- | --- | --- |
|  | **Maerl** | **Rock** | **Sand** | **Sand - Maerl** | **Rock - Maerl** | **Sand - Rock** |
| *Ablennes hians* | 0 (0) | 0.01 (0.1) | 0 (0) | 0, p=0.76 | 0, p=0.52 | 0, p=0.7 |
| *Abudefduf hoefleri* | 0.05 (0.55) | 0.08 (0.4) | 0.02 (0.18) | 0, p=1 | 0.003, p<0.01 | 0.004, p<0.01 |
| *Abudefduf saxatilis* | 0.09 (1) | 0.4 (1.17) | 0.47 (3.59) | 0, p=1 | 0.01, p<0.01 | 0.011, p<0.01 |
| *Abudefduf taurus* | 0.01 (0.09) | 0 (0) | 0.01 (0.07) | 0, p=0.95 | 0.001, p=0.03 | 0.001, p<0.01 |
| *Acanthocybium solandri* | 0.01 (0.09) | 0 (0) | 0 (0) | 0, p=0.76 | 0, p=0.53 | 0, p=0.71 |
| *Acanthostracion guineensis* | 0.02 (0.18) | 0 (0) | 0.01 (0.1) | 0.001, p=0.33 | 0, p=0.89 | 0, p=0.89 |
| *Acanthostracion notacanthus* | 0.08 (0.38) | 0.13 (0.42) | 0.11 (0.45) | 0.015, p=0.02 | 0.004, p=1 | 0.005, p=1 |
| *Acanthurus monroviae* | 0.92 (3.25) | 1.81 (3.95) | 0.68 (3.5) | 0.018, p=1 | 0.028, p<0.01 | 0.034, p<0.01 |
| *Alectis alexandrina* | 0.01 (0.09) | 0.02 (0.15) | 0.01 (0.1) | 0.003, p=0.18 | 0.001, p=0.96 | 0.001, p=0.98 |
| *Alectis ciliaris* | 0.01 (0.09) | 0.01 (0.1) | 0.04 (0.24) | 0.002, p=0.54 | 0, p=0.99 | 0.001, p=0.95 |
| *Aluterus heudelotii* | 0.03 (0.2) | 0.02 (0.15) | 0.04 (0.28) | 0.004, p=0.05 | 0.001, p=0.97 | 0.001, p=0.99 |
| *Aluterus scriptus* | 0.12 (0.45) | 0.08 (0.37) | 0.07 (0.34) | 0.009, p<0.01 | 0.005, p=0.94 | 0.004, p=1 |
| *Apsilus fuscus* | 0.02 (0.13) | 0.02 (0.21) | 0.04 (0.45) | 0.001, p=0.51 | 0.001, p=0.07 | 0.001, p=0.32 |
| *Ariosoma balearicum* | 0 (0) | 0.06 (0.62) | 0.04 (0.51) | 0.002, p=0.6 | 0, p=0.89 | 0.001, p=0.87 |
| *Atherina lopeziana* | 0 (0) | 0 (0) | 0.01 (0.14) | 0.001, p=0.77 | 0, p=0.52 | 0, p=0.7 |
| *Aulostomus strigosus* | 0.07 (0.4) | 0.18 (0.64) | 0.1 (0.48) | 0.003, p=1 | 0.008, p<0.01 | 0.008, p<0.01 |
| *Auxis rochei* | 0 (0) | 0.01 (0.1) | 0 (0) | 0, p=0.72 | 0, p=0.52 | 0, p=0.71 |
| *Auxis thazard* | 0.07 (0.5) | 0 (0) | 0 (0) | 0.001, p=0.42 | 0.001, p=0.27 | 0.001, p=0.38 |
| *Balistes capriscus* | 0.15 (0.88) | 0.03 (0.18) | 0.14 (0.56) | 0.014, p=0.11 | 0.002, p=1 | 0.005, p=1 |
| *Balistes punctatus* | 0.43 (0.85) | 0.63 (1.11) | 0.34 (0.75) | 0.03, p<0.01 | 0.018, p=1 | 0.022, p=0.9 |
| *Bodianus pulchellus* | 0.03 (0.2) | 0.09 (0.46) | 0.02 (0.12) | 0, p=1 | 0.003, p<0.01 | 0.003, p<0.01 |
| *Bodianus speciosus* | 0.22 (0.6) | 0.43 (0.86) | 0.3 (0.78) | 0.009, p=1 | 0.019, p<0.01 | 0.021, p<0.01 |
| *Boops boops* | 0 (0) | 0.01 (0.1) | 0.05 (0.72) | 0.001, p=0.09 | 0, p=0.69 | 0, p=0.91 |
| *Bothus sp.* | 0.21 (0.5) | 0.24 (0.54) | 0.32 (0.64) | 0.034, p<0.01 | 0.005, p=1 | 0.014, p=1 |
| *Cantherhines macrocerus* | 0.01 (0.09) | 0 (0) | 0 (0) | 0, p=0.78 | 0, p=0.1 | 0, p<0.01 |
| *Cantherhines pardalis* | 0.01 (0.09) | 0 (0) | 0.03 (0.32) | 0.001, p=0.43 | 0.001, p=0.24 | 0.001, p=0.34 |
| *Cantherhines pullus* | 1.26 (4.99) | 1.46 (5.27) | 1 (4.36) | 0.032, p<0.01 | 0.026, p=0.69 | 0.034, p<0.01 |
| *Canthidermis sufflamen* | 0.05 (0.29) | 0.08 (0.37) | 0.01 (0.1) | 0.004, p<0.01 | 0.003, p=0.29 | 0.002, p=0.7 |
| *Canthigaster supramacula* | 0.22 (0.72) | 0.44 (0.79) | 0.26 (0.82) | 0.007, p=1 | 0.019, p<0.01 | 0.022, p<0.01 |
| *Caranx bartholomaei* | 1.82 (4.47) | 1.63 (5.33) | 1.08 (2.97) | 0.047, p<0.01 | 0.024, p=1 | 0.03, p=1 |
| *Caranx crysos* | 1.27 (3.65) | 1.48 (4.84) | 1.3 (2.88) | 0.057, p<0.01 | 0.02, p=1 | 0.023, p=1 |
| *Caranx fischeri* | 0.01 (0.09) | 0 (0) | 0.01 (0.07) | 0.001, p=0.09 | 0, p=0.77 | 0, p=0.92 |
| *Caranx hippos* | 0.07 (0.29) | 0.28 (1) | 0.24 (1.97) | 0.011, p=0.73 | 0.005, p=1 | 0.008, p=0.99 |
| *Caranx latus* | 0.07 (0.48) | 0 (0) | 0.04 (0.44) | 0.003, p=0.39 | 0.001, p=0.85 | 0.002, p=0.79 |
| *Caranx lugubris* | 0.03 (0.2) | 0.02 (0.15) | 0.02 (0.14) | 0.004, p=0.3 | 0.001, p=0.98 | 0.001, p=0.98 |
| *Cephalopholis nigri* | 0.14 (0.55) | 0.6 (1.05) | 0.25 (0.78) | 0.002, p=1 | 0.021, p<0.01 | 0.025, p<0.01 |
| *Cephalopholis taeniops* | 0.42 (1.22) | 0.53 (1.15) | 0.25 (0.87) | 0.008, p=1 | 0.018, p<0.01 | 0.02, p<0.01 |
| *Chaetodon robustus* | 0.11 (0.45) | 0.11 (0.43) | 0.09 (0.39) | 0.004, p=0.95 | 0.007, p<0.01 | 0.007, p<0.01 |
| *Chilomycterus reticulatus* | 0.01 (0.09) | 0 (0) | 0.01 (0.1) | 0, p=0.66 | 0.001, p=0.12 | 0.001, p=0.16 |
| *Chilomycterus spinosus mauretanicus* | 0.05 (0.22) | 0.02 (0.15) | 0.03 (0.17) | 0.007, p<0.01 | 0.002, p=0.99 | 0.001, p=1 |
| *Chromis cadenati* | 0.38 (4.11) | 0 (0) | 0 (0) | 0.001, p=0.77 | 0, p=0.58 | 0.001, p=0.71 |
| *Chromis multilineata* | 5.01 (22.03) | 11.53 (29.27) | 4.16 (16.04) | 0.004, p=1 | 0.041, p<0.01 | 0.047, p<0.01 |
| *Cirrhitus atlanticus* | 0.12 (0.39) | 0.25 (0.54) | 0.12 (0.46) | 0, p=1 | 0.015, p<0.01 | 0.017, p<0.01 |
| *Clepticus africanus* | 0.14 (1.38) | 0.95 (4.6) | 0.36 (3.39) | 0, p=1 | 0.006, p<0.01 | 0.006, p<0.01 |
| *Coris atlantica* | 0.36 (1.05) | 0.9 (1.87) | 0.42 (1.48) | 0.006, p=1 | 0.024, p<0.01 | 0.028, p<0.01 |
| *Dactylopterus volitans* | 0.41 (0.91) | 0.22 (0.66) | 0.35 (0.74) | 0.04, p<0.01 | 0.014, p=1 | 0.009, p=1 |
| *Decapterus macarellus* | 2.57 (15.49) | 1.06 (6.1) | 2.59 (22.8) | 0.013, p=0.15 | 0.004, p=0.99 | 0.006, p=0.99 |
| *Decapterus punctatus* | 0.18 (1.44) | 0 (0) | 0.86 (6.23) | 0.008, p<0.01 | 0.003, p=0.89 | 0.002, p=1 |
| *Diodon holocanthus* | 0.02 (0.13) | 0.01 (0.1) | 0.04 (0.19) | 0.006, p<0.01 | 0.002, p=0.83 | 0, p=1 |
| *Diodon hystrix* | 0.01 (0.09) | 0.05 (0.23) | 0.02 (0.12) | 0.001, p=0.99 | 0.002, p<0.01 | 0.002, p<0.01 |
| *Echeneis naucrates* | 0.14 (0.8) | 0.09 (0.28) | 0.01 (0.07) | 0.007, p<0.01 | 0.004, p=0.84 | 0.003, p=0.97 |
| *Echidna peli* | 0 (0) | 0.05 (0.27) | 0.01 (0.07) | 0, p=0.99 | 0.002, p<0.01 | 0.002, p<0.01 |
| *Elagatis bipinnulata* | 0.07 (0.28) | 0.05 (0.43) | 0.06 (0.28) | 0.009, p=0.04 | 0.003, p=1 | 0.004, p=1 |
| *Enchelycore nigricans* | 0.04 (0.38) | 0.12 (0.51) | 0.05 (0.24) | 0.001, p=1 | 0.006, p<0.01 | 0.006, p<0.01 |
| *Ephippus goreensis* | 0 (0) | 0.01 (0.1) | 0 (0) | 0, p=0.76 | 0, p=0.15 | 0, p<0.01 |
| *Epinephelus adscensionis* | 0.04 (0.24) | 0.03 (0.18) | 0.01 (0.1) | 0.001, p=1 | 0.002, p<0.01 | 0.003, p<0.01 |
| *Epinephelus aeneus* | 0 (0) | 0.01 (0.1) | 0.01 (0.07) | 0.001, p=0.23 | 0, p=0.77 | 0, p=0.91 |
| *Epinephelus costae* | 0.02 (0.18) | 0 (0) | 0 (0) | 0, p=0.75 | 0, p=0.52 | 0, p=0.71 |

**S11 Table (cont.)**: SIMPER pairwise comparisons of habitat types.

| **Species** | **Mean MaxN (SD)** | | | **Average contribution to dissimilarities and significance** | | |
| --- | --- | --- | --- | --- | --- | --- |
|  | **Maerl** | **Rock** | **Sand** | **Sand - Maerl** | **Rock - Maerl** | **Sand - Rock** |
| *Eucinostomus melanopterus* | 0 (0) | 0.02 (0.21) | 0 (0) | 0.001, p=0.77 | 0, p=0.52 | 0, p=0.71 |
| *Fistularia tabacaria* | 0.07 (0.26) | 0.1 (0.57) | 0.07 (0.31) | 0.008, p<0.01 | 0.006, p=0.45 | 0.003, p=1 |
| *Gnatholepis thompsoni* | 0 (0) | 0 (0) | 0.01 (0.14) | 0, p=0.77 | 0, p=0.52 | 0, p=0.71 |
| *Gobius rubropunctatus* | 0 (0) | 0 (0) | 0.03 (0.19) | 0.001, p=0.79 | 0.001, p=0.36 | 0.001, p=0.21 |
| *Gymnothorax afer* | 0 (0) | 0.02 (0.15) | 0.02 (0.12) | 0.001, p=0.84 | 0.001, p=0.22 | 0.001, p=0.12 |
| *Gymnothorax vicinus* | 0.08 (0.4) | 0.1 (0.36) | 0.06 (0.31) | 0.002, p=1 | 0.006, p<0.01 | 0.006, p<0.01 |
| *Heteroconger longissimus* | 0 (0) | 0.54 (4.88) | 0.59 (4.18) | 0.007, p=0.04 | 0.001, p=0.99 | 0.002, p=0.99 |
| *Heteropriacanthus cruentatus* | 0 (0) | 0.03 (0.31) | 0.02 (0.12) | 0, p=1 | 0.001, p<0.01 | 0.001, p<0.01 |
| *Hippocampus algiricus* | 0 (0) | 0 (0) | 0.01 (0.07) | 0, p=0.77 | 0.001, p=0.22 | 0.001, p<0.01 |
| *Holacanthus africanus* | 0.22 (0.68) | 0.48 (0.88) | 0.2 (0.68) | 0.002, p=1 | 0.02, p<0.01 | 0.023, p<0.01 |
| *Holocentrus adscensionis* | 0.55 (2.86) | 0.4 (1.22) | 0.11 (0.57) | 0.002, p=1 | 0.013, p<0.01 | 0.014, p<0.01 |
| *Hypleurochilus aequipinnis* | 0 (0) | 0 (0) | 0.02 (0.22) | 0.001, p=0.78 | 0, p=0.53 | 0, p=0.71 |
| *Katsuwonus pelamis* | 0 (0) | 0.01 (0.1) | 0 (0) | 0, p=0.78 | 0, p=0.09 | 0, p<0.01 |
| *Kyphosus incisor* | 0.17 (1.83) | 0.17 (1.28) | 0.02 (0.23) | 0, p=1 | 0.003, p<0.01 | 0.003, p<0.01 |
| *Kyphosus sectatrix* | 0 (0) | 0.56 (4.19) | 0.01 (0.07) | 0, p=1 | 0.003, p<0.01 | 0.003, p<0.01 |
| *Labrisomus nuchipinnis* | 0.04 (0.27) | 0 (0) | 0.02 (0.16) | 0.001, p=0.87 | 0.002, p=0.05 | 0.002, p=0.02 |
| *Lagocephalus laevigatus* | 0.11 (0.56) | 0.01 (0.1) | 0.24 (2.61) | 0.01, p=0.08 | 0.002, p=1 | 0.003, p=1 |
| *Lethrinus atlanticus* | 0.58 (2.75) | 1.47 (4.55) | 0.75 (3.91) | 0.02, p=0.57 | 0.021, p=0.22 | 0.024, p<0.01 |
| *Lutjanus agennes* | 0.19 (0.99) | 0.85 (4.46) | 0.26 (2.12) | 0.005, p=1 | 0.011, p<0.01 | 0.013, p<0.01 |
| *Lutjanus dentatus* | 0.04 (0.2) | 0.14 (0.38) | 0.01 (0.1) | 0.001, p=1 | 0.005, p<0.01 | 0.006, p<0.01 |
| *Lutjanus endecacanthus* | 0.01 (0.09) | 0 (0) | 0.03 (0.19) | 0.001, p=0.84 | 0.002, p=0.38 | 0.002, p=0.15 |
| *Lutjanus fulgens* | 0.28 (1.4) | 0.69 (4) | 0.16 (1.44) | 0.001, p=1 | 0.009, p<0.01 | 0.01, p<0.01 |
| *Lutjanus goreensis* | 0.07 (0.49) | 0.08 (0.45) | 0.02 (0.18) | 0, p=1 | 0.003, p<0.01 | 0.003, p<0.01 |
| *Lutjanus griseus* | 0 (0) | 0 (0) | 0.02 (0.22) | 0, p<0.01 | 0, p=0.52 | 0, p=0.7 |
| *Megalops atlanticus* | 0 (0) | 0.01 (0.1) | 0 (0) | 0.001, p=0.77 | 0, p=0.52 | 0, p=0.7 |
| *Melichthys niger* | 0.12 (1.19) | 0.24 (1.22) | 0 (0) | 0.001, p=0.99 | 0.003, p<0.01 | 0.003, p<0.01 |
| *Microlipophrys velifer* | 0.01 (0.09) | 0 (0) | 0 (0) | 0, p<0.01 | 0, p=0.52 | 0, p=0.7 |
| *Microphis aculeatus* | 0 (0) | 0 (0) | 0.02 (0.14) | 0.003, p<0.01 | 0.001, p=0.76 | 0, p=0.99 |
| *Microspathodon frontatus* | 0.03 (0.37) | 0.31 (2.24) | 0.18 (1.32) | 0, p=1 | 0.004, p<0.01 | 0.005, p<0.01 |
| *Mulloidichthys martinicus* | 0.76 (3.04) | 1.76 (5.01) | 0.49 (1.79) | 0.005, p=1 | 0.022, p<0.01 | 0.025, p<0.01 |
| *Mullus surmuletus* | 0.03 (0.2) | 0 (0) | 0.04 (0.29) | 0.002, p=0.37 | 0.001, p=0.86 | 0.001, p=0.84 |
| *Muraena melanotis* | 0.09 (0.61) | 0.02 (0.15) | 0.03 (0.17) | 0.001, p=1 | 0.004, p<0.01 | 0.004, p<0.01 |
| *Muraena robusta* | 0.02 (0.13) | 0.01 (0.1) | 0 (0) | 0, p=0.99 | 0.001, p<0.01 | 0.001, p<0.01 |
| *Myrichthys pardalis* | 0 (0) | 0 (0) | 0.02 (0.14) | 0.001, p=0.66 | 0.001, p=0.49 | 0.001, p=0.36 |
| *Myripristis jacobus* | 0.08 (0.75) | 0.67 (3.36) | 0.09 (0.62) | 0, p=1 | 0.006, p<0.01 | 0.007, p<0.01 |
| *Nicholsina usta* | 0.02 (0.18) | 0 (0) | 0 (0) | 0, p=0.77 | 0, p=0.14 | 0, p<0.01 |
| *Ophichthus ophis* | 0.02 (0.13) | 0.03 (0.23) | 0.01 (0.1) | 0.003, p<0.01 | 0.001, p=0.87 | 0, p=0.99 |
| *Ophichthus rufus* | 0 (0) | 0 (0) | 0.01 (0.07) | 0.001, p=0.77 | 0, p=0.53 | 0, p=0.7 |
| *Ophioblennius atlanticus* | 0.12 (0.57) | 0.1 (0.36) | 0.02 (0.18) | 0, p=1 | 0.005, p<0.01 | 0.006, p<0.01 |
| *Pagrus caeruleostictus* | 0.25 (1.2) | 0.18 (0.85) | 0.21 (1.63) | 0.016, p<0.01 | 0.004, p=1 | 0.004, p=1 |
| *Pagrus pagrus* | 0.01 (0.09) | 0 (0) | 0 (0) | 0.001, p<0.01 | 0, p=0.53 | 0, p=0.71 |
| *Paraconger macrops* | 0 (0) | 0 (0) | 0.01 (0.1) | 0.001, p=0.65 | 0, p=0.77 | 0, p=0.91 |
| *Parakuhlia macrophthalmus* | 0 (0) | 0.01 (0.1) | 0 (0) | 0, p=0.77 | 0, p=0.07 | 0, p<0.01 |
| *Paranthias furcifer* | 20.54 (74.04) | 21.23 (39.56) | 11.24 (30.81) | 0.035, p=1 | 0.059, p<0.01 | 0.074, p<0.01 |
| *Phaeoptyx pigmentaria* | 0.01 (0.09) | 0 (0) | 0 (0) | 0.001, p<0.01 | 0, p=0.53 | 0, p=0.71 |
| *Pisodonophis semicinctus* | 0 (0) | 0.01 (0.1) | 0 (0) | 0, p<0.01 | 0, p=0.52 | 0, p=0.71 |
| *Pomadasys incisus* | 0.17 (1.83) | 0 (0) | 0 (0) | 0.001, p=0.77 | 0, p=0.61 | 0.001, p=0.56 |
| *Prionurus biafraensis* | 0.84 (6.81) | 4.33 (21.41) | 1.07 (7.68) | 0.001, p=1 | 0.015, p<0.01 | 0.017, p<0.01 |
| *Pseudupeneus prayensis* | 0.22 (0.84) | 1.16 (7.34) | 0.4 (1.81) | 0.013, p=0.87 | 0.014, p=0.47 | 0.016, p=0.07 |
| *Rypticus saponaceus* | 0.22 (0.54) | 0.16 (0.37) | 0.15 (0.37) | 0.012, p=0.79 | 0.014, p=0.19 | 0.016, p<0.01 |
| *Sardinella sp.* | 0 (0) | 0 (0) | 0.12 (1.25) | 0.001, p=0.66 | 0, p=0.78 | 0.001, p=0.77 |
| *Sargocentron hastatum* | 0.01 (0.09) | 0 (0) | 0 (0) | 0, p=0.78 | 0, p=0.09 | 0, p<0.01 |
| *Scarus hoefleri* | 0.09 (0.55) | 0.51 (1.1) | 0.24 (1.2) | 0.001, p=1 | 0.016, p<0.01 | 0.019, p<0.01 |
| *Scomber colias* | 0 (0) | 0 (0) | 0.01 (0.07) | 0, p=0.78 | 0, p=0.09 | 0, p<0.01 |
| *Scomberomorus tritor* | 0.03 (0.22) | 0.09 (0.32) | 0.08 (0.29) | 0.007, p=0.17 | 0.004, p=0.93 | 0.004, p=0.95 |
| *Scorpaena laevis* | 0.01 (0.09) | 0.01 (0.1) | 0 (0) | 0.001, p<0.01 | 0, p=0.52 | 0, p=0.92 |
| *Seriola rivoliana* | 0.07 (0.29) | 0.01 (0.1) | 0.01 (0.1) | 0.005, p=0.01 | 0.002, p=0.93 | 0.002, p=0.99 |
| *Serranus accraensis* | 0.02 (0.18) | 0 (0) | 0 (0) | 0.001, p<0.01 | 0, p=0.52 | 0, p=0.71 |
| *Serranus cabrilla* | 0.1 (0.47) | 0.09 (0.56) | 0.17 (0.77) | 0.012, p<0.01 | 0.003, p=1 | 0.004, p=1 |
| *Serranus pulcher* | 2.78 (6.72) | 1.09 (2.96) | 1.77 (4.19) | 0.106, p<0.01 | 0.04, p=1 | 0.009, p=1 |

**S11 Table (cont.)**: SIMPER pairwise comparisons of habitat types.

| **Species** | **Mean MaxN (SD)** | | | **Average contribution to dissimilarities and significance** | | |
| --- | --- | --- | --- | --- | --- | --- |
|  | **Maerl** | **Rock** | **Sand** | **Sand - Maerl** | **Rock - Maerl** | **Sand - Rock** |
| *Sparisoma choati* | 0.6 (1.47) | 1.78 (4.59) | 0.58 (3.14) | 0.011, p=1 | 0.028, p<0.01 | 0.035, p<0.01 |
| *Sparisoma rubripinne* | 0.04 (0.27) | 0.04 (0.29) | 0.01 (0.1) | 0.002, p=0.48 | 0.002, p=0.14 | 0.002, p=0.28 |
| *Sphoeroides marmoratus* | 0.18 (0.48) | 0.11 (0.4) | 0.24 (0.61) | 0.026, p<0.01 | 0.008, p=1 | 0.006, p=1 |
| *Sphyraena barracuda* | 0.12 (0.36) | 0.08 (0.27) | 0.11 (0.34) | 0.017, p<0.01 | 0.006, p=1 | 0.006, p=1 |
| *Spicara melanurus* | 0.07 (0.82) | 1.65 (7.25) | 0.31 (3.96) | 0.002, p=0.91 | 0.004, p<0.01 | 0.004, p=0.02 |
| *Spicara nigricauda* | 0 (0) | 0.12 (1.14) | 0.04 (0.39) | 0.001, p=0.68 | 0.001, p=0.13 | 0.001, p=0.12 |
| *Stegastes imbricatus* | 0.27 (0.96) | 0.92 (2.08) | 0.27 (0.96) | 0.002, p=1 | 0.02, p<0.01 | 0.023, p<0.01 |
| *Stephanolepis hispidus* | 0.06 (0.27) | 0.01 (0.1) | 0.05 (0.28) | 0.009, p<0.01 | 0.004, p=0.8 | 0.001, p=1 |
| *Thalassoma ascensionis* | 0.01 (0.09) | 0 (0) | 0 (0) | 0, p=0.77 | 0, p=0.15 | 0, p<0.01 |
| *Thalassoma newtoni* | 1.32 (3.31) | 1.74 (3.33) | 0.73 (1.86) | 0.013, p=1 | 0.036, p<0.01 | 0.045, p<0.01 |
| *Thalassoma pavo* | 0.02 (0.18) | 0.01 (0.1) | 0.02 (0.29) | 0, p=0.99 | 0.001, p<0.01 | 0.001, p<0.01 |
| *Trachinotus ovatus* | 0 (0) | 0.08 (0.73) | 0.18 (2.37) | 0.005, p=0.01 | 0.001, p=0.85 | 0.001, p=0.96 |
| *Trachinus lineolatus* | 0 (0) | 0 (0) | 0.01 (0.07) | 0, p=0.76 | 0, p=0.52 | 0, p=0.71 |
| *Uranoscopus polli* | 0 (0) | 0.01 (0.1) | 0 (0) | 0, p=0.74 | 0, p=0.52 | 0, p=0.71 |
| *Xyrichtys novacula* | 0.62 (1.57) | 0.44 (1.31) | 0.84 (1.81) | 0.044, p<0.01 | 0.013, p=1 | 0.014, p=1 |
