# Supplementary material for: Fish community composition in the tropical archipelago of São Tomé and Príncipe
Source: PLoS One. 2024 Nov 1;19(11):e0312849. doi: 10.1371/journal.pone.0312849 (PMC11530061; doi:10.1371/journal.pone.0312849)
Supplement: S1 File — (DOCX) [file pone.0312849.s019.docx]

# ADDITIONAL METHODOLOGICAL INFORMATION

## Sampling design

### Selection of sampling area

BRUVS sampling area was defined a priori, using bathymetric data from two digitised nautical charts (Instituto Hidrográfico Português, 1995) that were interpolated into a raster of ~120-metre resolution using Triangular Interpolation Network in QGIS (v. 3.28.1) (QGIS, 2023). Sampling area was defined as the area comprised between the shorelines of both islands and the 28-metre isobaths. In Príncipe, this area covered all coastal areas and spanned ~100 km^2^. In São Tomé, this was not possible due to logistical constrains, and only the Southern half of the island was selected for sampling, resulting on a sampling area of ~70 km^2^. This area was prioritised as it has been highlighted by local stakeholders as an important area for conservation and fisheries (Nuno et al., 2024). This Tinhosas islets (Fig 1, S1 Fig) were also sampled opportunistically and had a sampling area of 0.08 km^2^. Although they are part of Príncipe’s shelf, the Tinhosas were considered a third island category due to their isolation and higher ocean exposure (i.e., located at Príncipe’s shelf edge).

### Temporal and seasonal distribution of sampling

Sampling was conducted for two years (2018/2019 and 2019/2020) in Príncipe and for one year in São Tomé (2019/2020) since the project within which this sampling was conducted only started in the latter island in 2019/2020. To capture seasonal variations in our experimental design, sampling was stratified across two seasons: a cold-water season (July to September), locally known and hereafter referred to as gravana season; and a warmer water season (December to April) hereafter referred to as summer season (see S1 Table). Therefore, on each island, two sampling rounds (targeting the summer and gravana seasons) were conducted per sampling year. Due to COVID-19 restrictions, the 2020 summer sampling in Príncipe was curtailed in April and postponed to June-July 2020 (during the gravana season).

### Selection of sampling locations

Random sampling was chosen as an appropriate method for selecting the location of BRUVS deployments, since the lack of information on habitat distribution prevented the use of habitat-stratified sampling. Point location was changed on every season to implicitly capture a wider range of environmental conditions. On each sampling round, locations of BRUVS deployments were selected *a priori* using the tool “Create random points” of QGIS (QGIS, 2023), with a point density of approximately one point per 1.6 km^2^ (60 and 40 random points per sampling round in Príncipe and São Tomé, respectively). Since this algorithm resulted in substantial point clustering, to improve spatial balance, the sampling areas of each island were divided into equal area polygons of 15 km^2^ ± 3 km^2^ with 10 random points assigned to each polygon during each sampling season (S1 and S2 Fig). A minimum distance of 400 m between deployments was specified to avoid overlapping bait plumes of deployments conducted simultaneously and ensure independence of sampling (Whitmarsh et al., 2017).

Rocky habitats were less represented in the 2018/2019 random sampling round than other habitats due to their smaller extension. For this reason, in the 2019/2020 sampling, an additional 70 and 39 BRUVS were deployed on São Tomé and Príncipe, respectively, deliberately targeting rocky reef areas to increase the representation of this habitat class. Location of rocky reefs was determined with the help of artisanal fishers.

Finally, the Tinhosas Islets were sampled opportunistically, and alongside other research activities conducted in those islets, with three sampling points sampled three different times from 2018 to 2020.

## Video analysis and species identification

BRUVS recordings were analysed using VLC Media Player (v.2.1.3, [www.videolan.org/vlc](http://www.videolan.org/vlc)), and data was recorded using commercial spreadsheet software. For each species, the maximum number of individuals per frame (MaxN), was recorded alongside the time in the video at which they were observed (Whitmarsh et al., 2017). The same species was only recorded again if it was observed in a higher MaxN than the previous observation in the video. All teleost and elasmobranch species observed in BRUVS videos were recorded and identified to the lowest possible taxonomic level using existing species records and lists for the archipelago (Afonso et al., 1999; Wirtz et al., 2007; Vasco-Rodrigues et al., 2016) and regional species lists obtained from tools available at FishBase (Froese & Pauly, 2024).

## Definition of environmental variables

**Island** was defined as three categorical variables (**Príncipe**, **São Tomé** and the **Tinhosas**). While the Tinhosas islets are part of Príncipe’s shelf, they were considered a separate island category due to their remoteness and distinct environmental conditions (i.e. located at the edge of the shelf).

**Habitat type (rocky reef, sand, or maerl)** was qualitatively described from the field of view of BRUVS recordings. When two or more habitat types were present in the same recording, the deployment was classified based on the dominant habitat (i.e., the habitat covering over 50% of the field of view). Habitat types were classified as follows: **(1)** sandy bottoms, an open, uniform habitat dominated by sand; **(2)** maërl or rhodolith beds, locally known as “gla gla” and composed of biogenic structures formed by unattached, calcareous algae that form small globular structures over sandy bottoms; and **(3)** rocky reefs, rock platforms and boulder beds (Cowburn, 2018; Otero-Ferrer et al., 2020; Cosme De Esteban et al., 2023)

**Depth (metres)** was measured in the field using a handheld depth sounder (model: *Plastimo EchoTest II*, see Section 2.4 of article).

**Seabed slope (degrees)** was computed from a depth raster with a resolution of ~120m using the R package *raster* (Hijmans et al., 2020). This depth raster was the same used to define the boundaries of the sampling area (see section S1.1 of Supplementary Materials), which we obtained by interpolating depth points from two digitised nautical charts (Instituto Hidrográfico Português, 1995) into a raster using Triangular Interpolation Network in QGIS (v. 3.28.1) (QGIS, 2023).

**Distance to shore** was computed using the R package *geosphere* (Hijmans et al., 2022) and the shoreline was obtained from a shapefile of São Tomé and Príncipe obtained from <https://gadm.org/>.

**Intra-annual seasonality** (within years) was measured using ordinal dates. Due to logistical constraints, BRUVS sampling was conducted during the months of December to April and June to September (i.e., 10 different months were represented in our sample). Due to this ample variation, we considered ordinal date a more appropriate variable for capturing seasonality than using a categorical variable for season (e.g., “summer” vs “gravana”).

## Data analysis

### Information-Theoretic approach

We employed an information theoretic (IT) approach (Richards et al., 2011) to explore the effects of environmental variables (see S1.3) on diversity indicators (richness, abundance and evenness). We used (1) model selection approach to identify models that performed best in explaining variation in the data and (2) model averaging to obtain parameter estimates.

For each diversity indicator, models containing all possible combinations of effects were ranked using Akaike Information Criteria (AIC) using the function *MuMIn::dredge* in R (Bartoń, 2010). AIC is a metric of model performance, with lower AIC values indicating better performance. Best-performing models were selected through ΔAIC, which is the difference between the AIC of a given model and the AIC of the best performing model (i.e. the model with the lowest AIC value). Models with a ΔAIC < 6 were retained, as a threshold ΔAIC < 6 value is considered necessary to be 95% sure that the most parsimonious models are within the retained set of models (Richards et al., 2011). To reduce the retention of overly complex models, we excluded those models that were a more complicated version of any model within the retained set with a lower AIC value^[[1]](#footnote-1)^ (Burnham & Anderson, 2004). This approach infers low support for those parameters that are present in more complex models but are absent from simpler (nested) models with lower AIC (Richards et al., 2011).

The relative importance of each variable within the retained models was calculated using the function *MuMIn::sw* (Bartoń, 2010) in R, which sums the weighted AIC values of the models in which the variable is present (Burnham & Anderson, 2004). This metric of relative importance ranges from 0 to 1, with values closer to one indicating strong support for a given variable, and values closer to zero indicating less support.

Finally, we used model averaging to obtain parameter estimates and model predictions, using the function *MuMIn:: model.avg* (Bartoń, 2010) in R. Since we were interested in determining effect size (i.e. which factors have the strongest effect on the response variable), we introduced a parameter estimate and error of zero into those models in which a given parameter was absent. Parameter estimates are then averaged across models.

### Distance-Based Redundancy Analysis

Distance-Based Redundancy Analysis (db-RDA) is a constrained ordination method to explore the effect of a set of explanatory variables on a response matrix, namely non-Euclidean distances on a species versus site matrix (Borcard et al., 2011). Here, we used Bray-Curtis distances as they are more appropriate for modelling community count data, and we used the function vegan::dbrda (Oksanen et al., 2022). A db-RDA ordination creates a series of orthogonal axes (the “constrained axes”) which are linear combinations of the explanatory variables; each axis explaining, in successive order, the maximum possible amount of variation in the response matrix (Bakker, 2024). The coefficients of each variable on the constrained db-RDA axes therefore reflect the effect size of each variable on the variance explained by each axis (Borcard et al., 2011). To assess the significance of the resulting db-RDA ordination and constrained axes, we conducted an ANOVA-like permutation test using the function vegan::anova.cca (Oksanen et al., 2022). The amount of variation explained by the db-RDA ordination (also known as “constrained variation”) is measured by R^2^. Since R^2^ decreases with sample size and increases with the number of explanatory variables, we adjusted it using the function *vegan::RsquareAdj* (Oksanen et al., 2022).

### SIMilarity PERcentage analysis

To identify which species were driving dissimilarities across pairs of factors (i.e., habitats and islands), we used Similarity Percentages (SIMPER) using the function *vegan::simper* (Oksanen et al., 2022) in R. SIMPER is based on the decomposition of Bray-Curtis dissimilarity index and returns the contribution of each species to the average between-group Bray-Curtis dissimilarity.

SIMPER’s estimation of the contribution of each species to overall dissimilarities is not only driven by between-group differences, but also by variations in species abundance. Thus, highly variable species will be singled out by the method even if there are no significant differences across pairs of groups (Warton et al., 2012). Therefore, a permutation test (n = 9999) built within the *vegan::simper* function was used to identify those species for which the differences among pairs of factors contributed significantly to overall dissimilarities (Oksanen et al., 2022).

## References

Afonso, P., Porteiro, F., Santos, R., Barreiros, J., Worms, J. & Wirtz, P. (1999). Coastal marine fishes of São Tomé Island (Gulf of Guinea). *ARQUIPÉLAGO - Revista da Universidade dos Açores*, 65–92.

Bakker, J.D. (2024). *Applied Multivariate Statistics in R*. University of Washington.

Bartoń, K. (2010). MuMIn: Multi-Model Inference. 1.47.5.

Borcard, D., Gillet, F. & Legendre, P. (2011). *Numerical Ecology with R*. Springer: New York, NY.

Burnham, K.P. & Anderson, D.R. (Eds.). (2004). *Model Selection and Multimodel Inference*. Springer: New York, NY.

Cosme De Esteban, M., Haroun, R., Tuya, F., Abreu, A.D. & Otero-Ferrer, F. (2023). Mapping marine habitats in the Gulf of Guinea: A contribution to the future establishment of Marine Protected Areas in Principe Island. *Regional Studies in Marine Science*, 57, 102742. https://doi.org/10.1016/j.rsma.2022.102742

Cowburn, B.C. (2018). Marine Habitats of Príncipe, Eastern Tropical Atlantic – Description and Map. Available at: https://omaliprincipe.weebly.com/uploads/2/5/6/2/25623460/mapping_report_bcowburn-compressed.pdf

Froese, R. & Pauly, D. (2024). FishBase. Available at: www.fishbase.org

Hijmans, R.J., Etten, J.V., Sumner, M., Cheng, J., Baston, D., Bevan, A., et al. (2020). Package ‘raster’.

Hijmans, R.J., Karney (GeographicLib), C., Williams, E. & Vennes, C. (2022). geosphere: Spherical Trigonometry.

Instituto Hidrográfico Português. (1995). Missão Hidrográfica de Angola e São Tomé – 1962 charts.

Nuno, A., Madruga, L., Cameron, A., Airaud, F., Andrade, C., Nazaré, L., et al. (2024). Establishing a Marine Protected Area network using a Marine Spatial Planning approach: A reflection on practical challenges and opportunities for social–ecological integration. *Conservation Science and Practice*, (e13196), 1–17. https://doi.org/10.1111/csp2.13196

Oksanen, J., Simpson, G.L., Blanchet, F.G., Kindt, R., Legendre, P., Minchin, P.R., et al. (2022). vegan: Community Ecology Package.

Otero-Ferrer, F., Tuya, F., Bosch Guerra, N., Herrero-Barrencua, A., Abreu, A. & Haroun, R. (2020). Composition, structure and diversity of fish assemblages across seascape types at Príncipe, an understudied tropical island in the Gulf of Guinea (eastern Atlantic Ocean). *African Journal of Marine Science*, 42(4), 381–391. https://doi.org/10.2989/1814232X.2020.1826358

QGIS. (2023). QGIS. A Free and Open Source Geographic Information System.

Richards, S.A., Whittingham, M.J. & Stephens, P.A. (2011). Model selection and model averaging in behavioural ecology: the utility of the IT-AIC framework. *Behavioral Ecology and Sociobiology*, 65(1), 77–89. https://doi.org/10.1007/s00265-010-1035-8

Vasco-Rodrigues, N., Fontes, J. & Bertoncini, Á.A. (2016). Ten new records of marine fishes for São Tomé, West Africa. *Acta Ichthyologica et Piscatoria*, 46(2), 123–129. https://doi.org/10.3750/AIP2016.46.2.09

Warton, D.I., Wright, S.T. & Wang, Y. (2012). Distance-based multivariate analyses confound location and dispersion effects. *Methods in Ecology and Evolution*, 3(1), 89–101. https://doi.org/10.1111/j.2041-210X.2011.00127.x

Whitmarsh, S.K., Fairweather, P.G. & Huveneers, C. (2017). What is Big BRUVver up to? Methods and uses of baited underwater video. *Reviews in Fish Biology and Fisheries*, 27(1), 53–73. https://doi.org/10.1007/s11160-016-9450-1

Wirtz, P., Ferreira, C.E.L., Floeter, S.R., Fricke, R., Gasparini, J.L., Iwamoto, T., et al. (2007). Coastal fishes of São Tomé and Príncipe islands, Gulf of Guinea (Eastern Atlantic Ocean) - An update. *Zootaxa*, (1523), 1–48. https://doi.org/10.11646/zootaxa.1523.1.1

1. For example, if *Model 1* contains the same parameters as *Model 2* plus some additional ones, then *Model 1* is said to be a more complex version of *Model 2*, and *Model 2* is said to be nested within *Model 1*. If *Model 2* (nested) attains lower AIC than *Model 1* (more complex), then *Model 1* is removed from the retained set. [↑](#footnote-ref-1)
