# Supplementary material for: Fish community composition in the tropical archipelago of São Tomé and Príncipe
Source: PLoS One. 2024 Nov 1;19(11):e0312849. doi: 10.1371/journal.pone.0312849 (PMC11530061; doi:10.1371/journal.pone.0312849)
Supplement: S2 File — (DOCX) [file pone.0312849.s020.docx]

# S2. SUMMARY OF DEPLOYMENTS AND ENVIRONMENTAL CONDITIONS

On **Príncipe**, a total of 326 BRUVS were deployed from July 2018 to July 2020. Of these, 63 were excluded from further analyses due to data loss (n=30), camera/battery failure (n=22), low visibility (n=8) and loss of BRUVS (n=3). A total of 263 BRUVS deployments were therefore retained for further analysis, of which 150 were deployed during the gravana season and 113 during the summer season. In terms of environmental and physical characteristics, BRUVS were deployed in waters between 2.0 and 30.8 metres in depth (mean depth: 17.5 metres, SD: 6.9 metres, Figure S3), and between 7 and 5,311 metres offshore (mean offshore distance: 756 metres, SD: 852 metres), with a minimum and maximum slope of 0.1 degrees and 10.7 degrees, respectively (mean slope: 1.6, SD: 1.7). In terms of benthic habitat types, 133 BRUVs were deployed on sandy flats, 79 on maerl beds, and 51 on rocky reefs. On the **Tinhosas** islets, the same three locations were sampled three times (December 2018, July 2019, and March 2020) (Figure S1). Three deployments were excluded due to data loss, and six were retained for further analysis. The seabed around the Tinhosas Islets is a rocky reef environment, and BRUVS were deployed in waters between 14.7 and 23.0 metres (mean depth: 17.9 metres, SD: 3.0) and between 35 and 76 metres offshore (mean offshore distance: 57 metres, SD: 18 metres), with a minimum slope of 7.0 degrees and a maximum slope of 12.5 degrees (mean slope: 10.6, SD: 2.7). On **São Tomé**, a total of 163 BRUVS were deployed from September 2019 to February 2020, of which 15 were excluded from further analysis due to camera/battery failure (n=13), low visibility (n=1) and loss of BRUVs (n=1). A total of 148 deployments were therefore retained for further analysis, of which 74 were deployed during the gravana season and 74 during the summer season. In terms of environmental and physical characteristics, BRUVS were deployed in waters between 3.6 and 33.8 metres in depth (mean depth: 18.8 metres, SD: 7.0, Figure S3), and between 11 and 5,088 metres from the shore (mean offshore distance: 635 metres, SD: 1073), with a minimum slope of 0.15 degrees and maximum slope of 14 degrees (mean slope: 2.6 degrees, SD: 2.6 degrees). In terms of habitat composition, 71 BRUVs were deployed on sandy flats, 41 on maerl beds, and 36 on rocky reefs.
