# Supplementary material for: Fish community composition in the tropical archipelago of São Tomé and Príncipe
Source: PLoS One. 2024 Nov 1;19(11):e0312849. doi: 10.1371/journal.pone.0312849 (PMC11530061; doi:10.1371/journal.pone.0312849)
Supplement: Attachment — Submitted filename: Review.docx [file pone.0312849.s022.docx]

This is well-written manuscript describing assemblage of fish at Sao Tome and Pricipe in the Gulf of Guinea. The research approach and findings are not particularly novel, but this represents valuable data in a location where fish data are missing.

The BRUV methodology is executed very well and so are the analyses. I have minor comments and edits.

Line 35: change “whose” to “where coastal…”

Line 70: the writing is generally good, however this sentence was jarring. “Commonly used monitoring techniques to assess the health …” should read “Common techniques used to monitor and assess the health …

Line 108 and 109: you mention large fluctuations in SST but the mean values are 26 and 27 between seasons. This is not large, but I suspect this is because it is a mean. Maybe you could report the range or standard error or just say there are fluctuations in temperature, not necessarily large.

Line 134: the [11] is somewhat outdated. I understand the work was undertaken in 2019/20 but you should use:

Langlois, T., Goetze, J., Bond, T., Monk, J., Abesamis, R.A., Asher, J., Barrett, N., Bernard, A.T.F., Bouchet, P.J., Birt, M.J., Cappo, M., Currey‐Randall, L.M., Driessen, D., Fairclough, D. V., Fullwood, L.A.F., Gibbons, B.A., Harasti, D., Heupel, M.R., Hicks, J., Holmes, T.H., Huveneers, C., Ierodiaconou, D., Jordan, A., Knott, N.A., Lindfield, S., Malcolm, H.A., McLean, D., Meekan, M., Miller, D., Mitchell, P.J., Newman, S.J., Radford, B., Rolim, F.A., Saunders, B.J., Stowar, M., Smith, A.N.H., Travers, M.J., Wakefield, C.B., Whitmarsh, S.K., Williams, J., Harvey, E.S., 2020. A field and video annotation guide for baited remote underwater stereo‐video surveys of demersal fish assemblages. Methods in Ecology and Evolution 11, 1401–1409. <https://doi.org/10.1111/2041-210X.13470>

This paper is comprehensive and your methods are very well aligned.

Section 2.3 is great to see.

Line 160: analysis is not often started five minutes after landing on the seafloor but this is acceptable. Sometimes this is a period where you find the most cryptic species which are visible before the bait attending specie arrive. Something to consider in the future.

Line 165: just call it MaxN not maximum MaxN. MaxN is already the maximum.

Section 2.5: I wonder why you have not used year as a factor too. I understand that you added more deployments after not getting enough reef in the first year, but it would be interesting to see if anything changed between years. Habitat and depth is still going to drive everything.

Statistics: good!

Results: thorough and reporting all the metrics I like to see.

Line 398: change “that” to “than”.

Discussion: good. You have focusses on the ecology, mostly. I would like to see more around the fisheries. I know you have not measured any fish, but I believe you could comment on size classes and if you observed any larger fish deeper or further away.

Fig 3: Depth by Richness RI = 1? These plots look too perfect for a GAM. Having points scattered on the plots would provide the reader with confidence that they are correct. Also, consider plotting your season facet in a polarplot. This joins up each end – see examples in

Bond, T., Langlois, T.J., Partridge, J.C., Birt, M.J., Malseed, B.E., Smith, L., McLean, D.L., 2018. Diel shifts and habitat associations of fish assemblages on a subsea pipeline. Fisheries Research 206, 220–234. https://doi.org/10.1016/j.fishres.2018.05.011
