# Supplementary material for: Fish community composition in the tropical archipelago of São Tomé and Príncipe
Source: PLoS One. 2024 Nov 1;19(11):e0312849. doi: 10.1371/journal.pone.0312849 (PMC11530061; doi:10.1371/journal.pone.0312849)
Supplement: Attachment — Submitted filename: Response to reviewers.docx [file pone.0312849.s023.docx]

Manuscript **PONE-D-24-35103**

Response to Reviewers and editors

We wish to thank the editor and reviewers for giving us the opportunity to submit a revised draft of our manuscript (“Fish community composition in the tropical archipelago of São Tomé and Príncipe) for publication in PLOS ONE. We appreciate the time and effort that the reviewer and editor have dedicated to providing feedback, which has positively contributed to improving the paper. We have addressed the suggestions made by reviewers, which have been highlighted in a version of the manuscript with tracked changes. Below, we provide a point-by-point response, addressing the editor’s and the reviewer’s specific comments.

With best wishes,

Guillermo (on behalf of all authors)

# **Response to editor’s comments**

**Comment 1**. *Please ensure that your manuscript meets PLOS ONE's style requirements, including those for file naming. The PLOS ONE style templates can be found at* [*https://journals.plos.org/plosone/s/file?id=wjVg/PLOSOne_formatting_sample_main_body.pdf*](https://journals.plos.org/plosone/s/file?id=wjVg/PLOSOne_formatting_sample_main_body.pdf) *and* [*https://journals.plos.org/plosone/s/file?id=ba62/PLOSOne_formatting_sample_title_authors_affiliations.pdf*](https://journals.plos.org/plosone/s/file?id=ba62/PLOSOne_formatting_sample_title_authors_affiliations.pdf)

**Response 1**: Format of headings and subheadings, cross references to supplementary materials and captions have been updated to adjust PLOS ONE style templates.

**Comment 2**. *Please include a complete copy of PLOS’ questionnaire on inclusivity in global research in your revised manuscript. Our policy for research in this area aims to improve transparency in the reporting of research performed outside of researchers’ own country or community. The policy applies to researchers who have travelled to a different country to conduct research, research with Indigenous populations or their lands, and research on cultural artefacts. The questionnaire can also be requested at the journal’s discretion for any other submissions, even if these conditions are not met.  Please find more information on the policy and a link to download a blank copy of the questionnaire here: https://journals.plos.org/plosone/s/best-practices-in-research-reporting. Please upload a completed version of your questionnaire as Supporting Information when you resubmit your manuscript.*

**Response 2**: We have included a complete copy of PLOS’ questionnaire on inclusivity in global research.

**Comment 3**. *Thank you for stating the following financial disclosure:
“Fieldwork described here was undertaken within the project “Establishing a network of marine protected areas across São Tomé and Príncipe through a co-management approach” and was funded by Blue Action Fund and Arcadia Fund for Nature - a charitable fund of Lisbet Rausing and Peter Baldwin. Preliminary work was funded by the Darwin Initiative - a UK government grant scheme (Project 23–012), Forever Príncipe and Halpin Trust. G.P. acknowledges funding from Fundação para a Ciência e Tecnologia (FCT) doctoral grant nº UI/BD/151263/2021. A.N. acknowledges the support of the European Union's Horizon 2020 research and innovation programme under the Marie Skłodowska‐Curie grant agreement SocioEcoFrontiers No. 843865. K.M. acknowledges funding support from the Darwin Initiative (Project 23-011 and 26-014), Waterloo Foundation, and the Wildlife Conservation Society Congo Programme. G.P and T.C.L. acknowledge the support received from cE3c - Center for Ecology, Evolution and Environmental Changes through FCT’s strategic project UIDB/00329/2020 (doi: 10.54499/UIDB/00329/2020)”
Please state what role the funders took in the study.  If the funders had no role, please state: "The funders had no role in study design, data collection and analysis, decision to publish, or preparation of the manuscript."
If this statement is not correct you must amend it as needed.
Please include this amended Role of Funder statement in your cover letter; we will change the online submission form on your behalf.*

**Response 3**: Thank you for pointing this out. Funders had no role in the study.

The Role of Funder statement should read as:

- “*The funders* had *no role in study design, data collection and analysis, decision to publish, or preparation of the manuscript.*”

The full Financial Disclosure statement should read as:

- “*Fieldwork described here was undertaken within the project “Establishing a network of marine protected areas across São Tomé and Príncipe through a co-management approach” and was funded by Blue Action Fund and Arcadia Fund for Nature - a charitable fund of Lisbet Rausing and Peter Baldwin. Preliminary work was funded by the Darwin Initiative - a UK government grant scheme (Project 23–012), Forever Príncipe and Halpin Trust. G.P. acknowledges funding from Fundação para a Ciência e Tecnologia (FCT) doctoral grant nº UI/BD/151263/2021. A.N. acknowledges the support of the European Union's Horizon 2020 research and innovation programme under the Marie Skłodowska‐Curie grant agreement SocioEcoFrontiers No. 843865. K.M. acknowledges funding support from the Darwin Initiative (Project 23-011 and 26-014), Waterloo Foundation, and the Wildlife Conservation Society Congo Programme. G.P and T.C.L. acknowledge the support received from cE3c - Center for Ecology, Evolution and Environmental Changes through FCT’s strategic project UIDB/00329/2020 (doi: 10.54499/UIDB/00329/2020). The funders* had *no role in study design, data collection and analysis, decision to publish, or preparation of the manuscript.*”

**Comment 4**. Thank you for stating the following in the Acknowledgments Section of your manuscript:
“Fieldwork described here was undertaken within the project “Establishing a network of marine protected areas across São Tomé and Príncipe through a co-management approach” and was funded by Blue Action Fund and Arcadia Fund for Nature - a charitable fund of Lisbet Rausing and Peter Baldwin. Preliminary work was funded by the Darwin Initiative - a UK government grant scheme (Project 23–012), Forever Príncipe and Halpin Trust. G.P. acknowledges funding from Fundação para a Ciência e Tecnologia (FCT) doctoral grant nº UI/BD/151263/2021. A.N. acknowledges the support of the European Union's Horizon 2020 research and innovation programme under the Marie Skłodowska‐Curie grant agreement SocioEcoFrontiers No. 843865. K.M. acknowledges funding support from the Darwin Initiative (Project 23-011 and 26-014), Waterloo Foundation, and the Wildlife Conservation Society Congo Programme. G.P and T.C.L. acknowledge the support received from cE3c - Centre for Ecology, Evolution and Environmental Changes through FCT’s strategic project UIDB/00329/2020 (doi: 10.54499/UIDB/00329/2020). We further thank Fundação Príncipe’s marine team for their support during field work, especially marine guards Ibizaltino Ceita, Abduley de Pina, Pascoal Monteiro, Zelinho Maquengo and Carlos Sanches, and project coordinators Vanessa Schmitt and Jaconias Pereira. We also wish to thank the team that supported São Tomé’s sampling, including captain Juvêncio and crew members, as well as Bastien Loloum.”
We note that you have provided funding information that is currently declared in your Funding Statement. However, funding information should not appear in the Acknowledgments section or other areas of your manuscript. We will only publish funding information present in the Funding Statement section of the online submission form.
Please remove any funding-related text from the manuscript and let us know how you would like to update your Funding Statement. Currently, your Funding Statement reads as follows:
“Fieldwork described here was undertaken within the project “Establishing a network of marine protected areas across São Tomé and Príncipe through a co-management approach” and was funded by Blue Action Fund and Arcadia Fund for Nature - a charitable fund of Lisbet Rausing and Peter Baldwin. Preliminary work was funded by the Darwin Initiative - a UK government grant scheme (Project 23–012), Forever Príncipe and Halpin Trust. G.P. acknowledges funding from Fundação para a Ciência e Tecnologia (FCT) doctoral grant nº UI/BD/151263/2021. A.N. acknowledges the support of the European Union's Horizon 2020 research and innovation programme under the Marie Skłodowska‐Curie grant agreement SocioEcoFrontiers No. 843865. K.M. acknowledges funding support from the Darwin Initiative (Project 23-011 and 26-014), Waterloo Foundation, and the Wildlife Conservation Society Congo Programme. G.P and T.C.L. acknowledge the support received from cE3c - Centre for Ecology, Evolution and Environmental Changes through FCT’s strategic project UIDB/00329/2020 (doi: 10.54499/UIDB/00329/2020)”
Please include your amended statements within your cover letter; we will change the online submission form on your behalf.

**Response 4**: Thanks for pointing this out. Acknowledgements section has been updated accordingly.

**Comment 5**. When completing the data availability statement of the submission form, you indicated that you will make your data available on acceptance. We strongly recommend all authors decide on a data sharing plan before acceptance, as the process can be lengthy and hold up publication timelines. Please note that, though access restrictions are acceptable now, your entire data will need to be made freely accessible if your manuscript is accepted for publication. This policy applies to all data except where public deposition would breach compliance with the protocol approved by your research ethics board. If you are unable to adhere to our open data policy, please kindly revise your statement to explain your reasoning and we will seek the editor's input on an exemption. Please be assured that, once you have provided your new statement, the assessment of your exemption will not hold up the peer review process.

**Response 5**: Thank you for clarification. Both links provided in the data availability statement are active and data has already been uploaded, but it is embargoed until acceptance of the manuscript (<https://doi.org/10.5281/zenodo.13326736> and <https://github.com/gporrinos/BRUVS_SaoTomePrincipe_2018_2020>). Data will be immediately made available upon confirmation of acceptance.

**Comment 6**. We note that Figures 1, S1 and S2 in your submission contain [map/satellite] images which may be copyrighted. All PLOS content is published under the Creative Commons Attribution License (CC BY 4.0), which means that the manuscript, images, and Supporting Information files will be freely available online, and any third party is permitted to access, download, copy, distribute, and use these materials in any way, even commercially, with proper attribution. For these reasons, we cannot publish previously copyrighted maps or satellite images created using proprietary data, such as Google software (Google Maps, Street View, and Earth). For more information, see our copyright guidelines: http://journals.plos.org/plosone/s/licenses-and-copyright.

We require you to either (1) present written permission from the copyright holder to publish these figures specifically under the CC BY 4.0 license, or (2) remove the figures from your submission:

a. You may seek permission from the original copyright holder of Figures 1, S1 and S2 to publish the content specifically under the CC BY 4.0 license.

We recommend that you contact the original copyright holder with the Content Permission Form (http://journals.plos.org/plosone/s/file?id=7c09/content-permission-form.pdf) and the following text:

“I request permission for the open-access journal PLOS ONE to publish XXX under the Creative Commons Attribution License (CCAL) CC BY 4.0 (http://creativecommons.org/licenses/by/4.0/). Please be aware that this license allows unrestricted use and distribution, even commercially, by third parties. Please reply and provide explicit written permission to publish XXX under a CC BY license and complete the attached form.”

Please upload the completed Content Permission Form or other proof of granted permissions as an "Other" file with your submission.

In the figure caption of the copyrighted figure, please include the following text: “Reprinted from [ref] under a CC BY license, with permission from [name of publisher], original copyright [original copyright year].”

b. If you are unable to obtain permission from the original copyright holder to publish these figures under the CC BY 4.0 license or if the copyright holder’s requirements are incompatible with the CC BY 4.0 license, please either i) remove the figure or ii) supply a replacement figure that complies with the CC BY 4.0 license. Please check copyright information on all replacement figures and update the figure caption with source information. If applicable, please specify in the figure caption text when a figure is similar but not identical to the original image and is therefore for illustrative purposes only.

The following resources for replacing copyrighted map figures may be helpful:

USGS National Map Viewer (public domain): http://viewer.nationalmap.gov/viewer/

The Gateway to Astronaut Photography of Earth (public domain): http://eol.jsc.nasa.gov/sseop/clickmap/

Maps at the CIA (public domain): https://www.cia.gov/library/publications/the-world-factbook/index.html and https://www.cia.gov/library/publications/cia-maps-publications/index.html

NASA Earth Observatory (public domain): http://earthobservatory.nasa.gov/

Landsat: http://landsat.visibleearth.nasa.gov/

USGS EROS (Earth Resources Observatory and Science (EROS) Center) (public domain): http://eros.usgs.gov/#

Natural Earth (public domain): http://www.naturalearthdata.com/

**Response 6**: Thank you for pointing this out. We had used bathymetry data from two digitised nautical charts from “Instituto Hidrográfico Português”. Nevertheless, since we have not been able to obtain the copyright information for that data, we have substituted that data by the GEBCO bathymetry data in all figures. The following statement has been added to the S1 and S2 Figure legends to reflect the new data source: “(Bathymetric data: © GEBCO)”.

**Comment 7**. Please review your reference list to ensure that it is complete and correct. If you have cited papers that have been retracted, please include the rationale for doing so in the manuscript text, or remove these references and replace them with relevant current references. Any changes to the reference list should be mentioned in the rebuttal letter that accompanies your revised manuscript. If you need to cite a retracted article, indicate the article’s retracted status in the References list and also include a citation and full reference for the retraction notice.

**Response 7**: We have reviewed the reference list and have not found any reference that has been retracted.

# **RESPONSE TO REVIEWER #1’S COMMENTS**

**Comment 1**. This is well-written manuscript describing assemblage of fish at Sao Tome and Pricipe in the Gulf of Guinea. The research approach and findings are not particularly novel, but this represents valuable data in a location where fish data are missing.
The BRUV methodology is executed very well and so are the analyses. I have minor comments and edits.

**Response 1**: Thank you!

**Comment 2**. Line 35: change “whose” to “where coastal…”

**Response 2**: Thanks for the suggestion, text was changed accordingly.

**Comment 3**. Line 70: the writing is generally good, however this sentence was jarring. “Commonly used monitoring techniques to assess the health …” should read “Common techniques used to monitor and assess the health …

**Response 3**: Thanks for the suggestion, text was changed accordingly.

**Comment 4**. Line 108 and 109: you mention large fluctuations in SST but the mean values are 26 and 27 between seasons. This is not large, but I suspect this is because it is a mean. Maybe you could report the range or standard error or just say there are fluctuations in temperature, not necessarily large.

**Response 4**: Thank you for pointing this out! These values were a mistake, the actual values reported in the reference [19] were above 28 and below 23. This has been corrected in the main text.

**Comment 5**. Line 134: the [11] is somewhat outdated. I understand the work was undertaken in 2019/20 but you should use:
Langlois, T., Goetze, J., Bond, T., Monk, J., Abesamis, R.A., Asher, J., Barrett, N., Bernard, A.T.F., Bouchet, P.J., Birt, M.J., Cappo, M., Currey‐Randall, L.M., Driessen, D., Fairclough, D. V., Fullwood, L.A.F., Gibbons, B.A., Harasti, D., Heupel, M.R., Hicks, J., Holmes, T.H., Huveneers, C., Ierodiaconou, D., Jordan, A., Knott, N.A., Lindfield, S., Malcolm, H.A., McLean, D., Meekan, M., Miller, D., Mitchell, P.J., Newman, S.J., Radford, B., Rolim, F.A., Saunders, B.J., Stowar, M., Smith, A.N.H., Travers, M.J., Wakefield, C.B., Whitmarsh, S.K., Williams, J., Harvey, E.S., 2020. A field and video annotation guide for baited remote underwater stereo‐video surveys of demersal fish assemblages. Methods in Ecology and Evolution 11, 1401–1409. https://doi.org/10.1111/2041-210X.13470
This paper is comprehensive and your methods are very well aligned.

**Response 5**: Thanks for providing this reference. We have substituted the Whitmarsh et al (2017) reference by this one in all instances.

**Comment 6**. Section 2.3 is great to see.

**Response 6**. Thanks!

**Comment 7**. Line 160: analysis is not often started five minutes after landing on the seafloor but this is acceptable. Sometimes this is a period where you find the most cryptic species which are visible before the bait attending specie arrive. Something to consider in the future.

**Response 7**. Thanks for the suggestion! We used a 5-min buffer to avoid interferences with the boat and to account for sediment disturbed following deployment, but we will certainly consider your point on cryptic species observations in the future.

**Comment 8**. Line 162: use original cite for MaxN Priede papers and Cappo papers. see BRUV paper suggested above for more detail.

**Response 8**. Thanks for providing the references. They have been changed accordingly:

32.	Priede IG, Bagley PM, Smith A, Creasey S, Merrett NR. Scavenging deep demersal fishes of the Porcupine Seabight, north-east Atlantic: observations by baited camera, trap and trawl. J Mar Biol Assoc U K. 1994;74: 481–498. doi:10.1017/S0025315400047615

33.	Cappo M, Speare P, De’ath G. Comparison of baited remote underwater video stations (BRUVS) and prawn (shrimp) trawls for assessments of fish biodiversity in inter-reefal areas of the Great Barrier Reef Marine Park. J Exp Mar Biol Ecol. 2004;302: 123–152. doi:10.1016/j.jembe.2003.10.006

**Comment 9**. Line 165: just call it MaxN not maximum MaxN. MaxN is already the maximum.

**Response 9**. Thanks for the suggestion, text has been changed accordingly.

**Comment 10**. Section 2.5: I wonder why you have not used year as a factor too. I understand that you added more deployments after not getting enough reef in the first year, but it would be interesting to see if anything changed between years. Habitat and depth is still going to drive everything.

**Response 10**. Thanks for the suggestion. We did consider including year as a factor as we agree it would be interesting to investigate seasonal effects. However, given the unbalanced sample (sampling was conducted for two years in Príncipe, but only one year in São Tomé), we decided that our data were not sufficient to make robust conclusions about inter-annual seasonality. Nevertheless, we agree that it would be an important factor to consider, and we would be keen on including it in future analyses (sampling described in this article was the first of an ongoing monitoring of STP’s marine assemblages, which will be conducted every three years).

**Comment 11**. Statistics: good!
Results: thorough and reporting all the metrics I like to see.

**Response 11**. Thanks!

**Comment 12**. Line 398: change “that” to “than”.

**Response 12**. Thanks for spotting this, text has been changed accordingly.

**Comment 13**. Discussion: good. You have focusses on the ecology, mostly. I would like to see more around the fisheries. I know you have not measured any fish, but I believe you could comment on size classes and if you observed any larger fish deeper or further away.

**Response 13**. Thanks!

Whilst we agree this would be a valuable addition to the discussion, we don’t feel confident in making too many suggestive descriptions of the individuals seen, give that we used mono-BRUVS and therefore can’t estimate sizes. For you interest, we are also currently working on an additional fisheries landing paper that touches on these and other aspects, comparing catch across gears and islands.

**Comment 14**. Fig 3: Depth by Richness RI = 1? These plots look too perfect for a GAM. Having points scattered on the plots would provide the reader with confidence that they are correct. Also, consider plotting your season facet in a polarplot. This joins up each end – see examples in
Bond, T., Langlois, T.J., Partridge, J.C., Birt, M.J., Malseed, B.E., Smith, L., McLean, D.L., 2018. Diel shifts and habitat associations of fish assemblages on a subsea pipeline. Fisheries Research 206, 220–234. https://doi.org/10.1016/j.fishres.2018.05.011

**Response 14**. Thanks for your comments! Responses below

We defined relative importance (RI) presented in the figures as the sum of the weighted AICs of the models in which the predictor of interest was present, which was calculated using the *MuMIn::sw* function (formerly called *MuMIn::importance*). This measures the relative importance of a predictor *within the top model set*. We however acknowledge that this terminology may be confusing, as “relative importance” also refers to the contribution of an individual predictor to the deviance explained by a model. To better reflect our methods, we have substituted “relative importance” by “summed weights” (SW).

Lines 162 to 168 now read as “*Weighted averages of best performing models were used to estimate effects, substituting by zero when a predictor was not present in a model. The relative importance of predictors in the top model set was calculated by summing weighted AICs (Summed Weights, SW) across all models containing the variable of interest (e.g., if, in a top model set, predictor “A” is present in two models with weighted AICs = 0.36 and 0.37, then SW_A_ = 0.73). SW ranges from 0 to 1, with values closer to one indicating higher support and values closer to zero indicating lower support*”. Additionally, section “Species richness and relative abundance” of the results has been reworded to better reflect our method.

Additionally, we have calculated the contribution of predictors to the deviance explained by the individual models in the top model set, as we acknowledge that this is an important metric that we had not included in the original version. We have calculated this after Lai et al (2024) (<https://doi.org/10.1016/j.pld.2024.06.002>).

Lines 168 to 170 now read as “*The contribution of predictors to the deviance explained by the individual models in the model set was calculated after Lai et al [38] to account for the average shared variance of predictors with concurvity*”. Results have been included in Table S8 of supplementary materials and, when relevant, have been included in section “Species richness and relative abundance” of results.

Regarding depth, slope and distance to shore:

Thanks for noting this. In the previous version, depth and slope were fitted as linear effects (not splines) due to initial data interrogation suggesting such relationships between these variables. However, we acknowledge that we relied on assumptions, and not formal testing. Therefore, we have refitted the models with these variables included as thin plate splines. Depth and slope still show a linear effect (which we have noted in the main text), but distance to shore showed a non-linear effect in the relative abundance models.

Additionally, we have plotted the partial effect of smooths and residuals in S4 Fig, which is referenced in the caption of Fig S3 (line 278).

Thanks for the suggestion on the polar plots. We chose to keep the presentation of the results as they are, as these are most likely to be better interpreted by in-country stakeholders.
